# Systematic review and conceptualisation of disaffection for its accompaniment

**DOI:** 10.3389/fpsyg.2026.1677767

**Published:** 2026-04-23

**Authors:** José Víctor Orón Semper, Cristina Antón Rodríguez

**Affiliations:** 1 Facultad de Ciencias de la Salud, Universidad Francisco de Vitoria, Madrid, Spain; 2 Facultad de Medicina, Universidad Francisco de Vitoria, Madrid, Spain

**Keywords:** disaffection, Erick Fromm, purpose of life, stress, well-being

## Abstract

**Introduction:**

Disaffection is increasingly recognised as a psychological and relational process marked by the gradual loss of meaning in initially significant purposes, leading to relational deterioration and emotional distress. Although disaffection has been studied in fields such as education, political engagement, and healthcare, it remains poorly conceptualised as a distinct construct.

**Methods:**

This study addresses the definitional and disciplinary fragmentation surrounding disaffection by integrating a systematic review conducted in accordance with PRISMA 2020 guidelines with a theory-driven conceptual analysis. The review systematically identified and critically appraised empirical and theoretical uses of the term across three databases (Web of Science, Scopus, and PubMed) up to June 26, 2025, applying predefined inclusion and exclusion criteria regarding conceptual focus and study design, along with exclusion criteria related to methodological quality, assessed through Joanna Briggs Institute appraisal tools. The subsequent conceptual analysis, grounded in Erich Fromm’s theoretical framework, delineates defining attributes, underlying psychological processes, and relational dimensions in order to provide an operational framework for research and accompaniment. To further ensure methodological rigour and minimise potential bias, additional safeguards were implemented throughout the review process. The two-stage screening procedure (titles/abstracts followed by full-text assessment) strengthened selection consistency, while the application of JBI critical appraisal tools ensured design-appropriate quality evaluation. Structured data extraction procedures enhanced transparency and reduced the risk of reporting distortions. Regarding the conceptual component, the adoption of Fromm’s theoretical framework entails an interpretative positioning; this is explicitly acknowledged and theoretically justified to delimit the scope and implications of the analysis.

**Results:**

Sixty-seven studies were included, most from European contexts and covering political disaffection, disengagement in education, relational estrangement, and burnout in healthcare professions. Disaffection emerged as a multidimensional process with emotional (e.g., frustration, cynicism), behavioural (e.g., withdrawal, detachment, automatism), and cognitive (e.g., distrust, disidentification) components. The analysis highlights disaffection as distinct from related conditions such as depression, anxiety, or burnout, emphasising its specific relational and existential dimensions.

**Discussion:**

This review and conceptual analysis propose that disaffection is a progressive relational deterioration stemming from the perceived loss of meaning in commitments and interpersonal bonds, often leading to withdrawal into individualistic self-protection. The study provides an operationalisation of disaffection that may guide the development of instruments to detect early signs and inform preventive and reparative interventions. Limitations include reliance on Fromm’s theoretical perspective and the absence of validated empirical measures. Further empirical research is needed to test the proposed conceptualisation and its applicability in clinical and educational contexts. In conclusion, this study conceptualises disaffection as a distinct relational–existential process and provides an operational framework to guide early detection and humanising accompaniment across clinical, educational and organisational contexts.

## 1 Introduction and methods

Accompaniment – which may take diverse forms – seeks to offer help to the person in their individual, relational and work processes (Vasan et al., 2017; Serrano García et al., 2024). The nature of this help will depend on the person’s circumstances and the challenges they are facing. At times, accompaniment might be required to deal with a specific event and as such will be temporary; addressing an identified challenge or relational issue. At other times, there are cases when persons require a long-term accompaniment, which does not refer to a specific event but rather to a more complex ongoing situation. It is the latter type of case which we will deal with in this paper.

Disaffection is approached here as a transversal phenomenon that unfolds across diverse life domains and developmental stages, characterised by the progressive deterioration of initially meaningful relational commitments. Examining this trajectory clarifies the need for a unified conceptual framework capable of guiding appropriate forms of accompaniment.

We focus on situations in which individuals begin with enthusiasm and confidence in participating in a project experienced as valuable and meaningful, yet progressively encounter repeated and disappointing contrasts with reality that undermine their capacity to generate novelty. As this process unfolds, the relationships that once sustained the initial sense of purpose become reshaped by short-term, individualistic, and conformist orientations aimed merely at adapting to environmental demands. Clarifying this phenomenon is therefore essential in order to understand its internal dynamics and to determine how accompaniment can respond in a theoretically grounded and practically effective manner.

The phenomenon of “disaffection” is a complex one which pervades all areas of life and could be considered an “umbrella term.” The experience of disaffection can be recognised in the student who starts school with enthusiasm before demotivation takes its toll (Asensio et al., 2023). Similarly, it can be recognised in a person who starts a professional career with dreams of doing great things and then feels deflated when these great aspirations are at odds with reality (Díaz et al., 2010; Manzano, 2002; Pascual Jimeno and Conejero López, 2015; Sánchez Llull et al., 2015). Political corruption and unfulfilled promises generate citizen disaffection and discourage adherents, thus eroding democratic trust (Jennings et al., 2016; Rivers, 2021; Clarke et al., 2018; Ahmad et al., 2024). Employees experience initial enthusiasm, followed by disillusionment and job cynicism as they regulate emotions and cope with organisational reality (Lee et al., 2020; Boswell et al., 2005), a similar process occurs in public administration (Marsollier, 2019).

Progressive disillusionment is a phenomenon documented in multiple fields where initial motivation diminishes or disappears due to psychological and structural factors. In scientific and academic research, performance pressure and emotional overload lead to burnout and cynicism towards one’s work (Heo et al., 2020). In artistic creativity and production, initial enthusiasm is affected by social constraints and external expectations, leading to loss of motivation (Lee and Cho, 2019). In sports, high demand and competitive stress contribute to burnout syndrome, affecting the performance and well-being of athletes (Li et al., 2022). In the religious and vocational context, emotional burnout and faith crisis lead to a progressive loss of initial commitment (Fulmer 2023; Upenieks 2024). Finally, in interpersonal relationships and marriage, burnout and adjustment to living together can reduce marital satisfaction over time (Lavner et al., 2018). The areas to which this extends can be many and varied, and have been explored in disciplines ranging from philosophy to sociology (e.g., Le Bretón, 2023).

The above enables us to appreciate the relevance of disaffection because of both its impact across different areas of life and its “transitional” character. It involves an experience through which individuals move from the initial states, where the problem has not fully appeared, to situations which may already be pathological. Therefore, the term disaffection refers more to a process than to a static state. Elucidating the psychological processes that constitute this phenomenon could be helpful for accompaniment. Its early detection would enable practitioners to accompany individuals in the initial stages of disaffection, when the condition is still not severe and more resources are available for intervention.

Besides its usefulness for accompaniment, the identification of disaffection also concerns health. If it is not addressed, it could lead to either pathological outcomes or to a condition of helplessness or ego-weakness that diminishes the person’s resilience when faced with stress. Early intervention would thus have both a preventive dimension and a restorative potential. We hypothesise that a disaffected person, in comparison with someone who has developed a meaningful life purpose, has fewer psychological resources to confront stress. This might partially explain why the same stressful experience may be perceived as tolerable by one individual and as toxic by another.

The objective of this article is to offer a comprehensive conceptual characterisation of disaffection that may serve both as a framework for accompanying disaffected individuals and as a foundation for the future development of psychometric instruments to identify and assess this phenomenon. Similar conceptual efforts have been undertaken in psychology and psychiatry to understand how individuals position themselves in relation to certain experiential states in order to enable effective accompaniment and to anticipate trajectories that may culminate in psychopathology. In such approaches, an initial experiential condition—whether episodic or constitutive of a broader life orientation—may evolve negatively when integration fails. A relevant example is the concept of “embitterment,” introduced by Michael Linden to describe the psychological processes that arise when individuals are unable to creatively integrate experiences of perceived injustice, thereby developing specific maladaptive patterns (Linden, 2011; Linden et al., 2008; Linden and Muschalla, 2011). In a comparable yet distinct manner, the present study seeks to delineate the psychological processes that emerge when a person undergoes a progressive shift from an initially meaningful life purpose—experienced as valuable and orienting—to a condition in which that value is perceived as lost, and engagement becomes reduced to the management of external or internal demands centred on mere well-being or self-centred pursuits.

To achieve our purpose, the following itinerary was followed:

First, a description of the term disaffection was sought in the existing academic literature. To this end, a systematic review (Higgins, 2019) was carried out and the results were ordered according to a classification typical of the social sciences into emotional, behavioural, and cognitive components about the term (Allport, 1935; Rosenberg and Hovland, 1960; Briñol and Petty, 2006). This made it possible to arrive at a phenomenological description of disaffection.
Secondly, a definition of the term was sought that emphasises the relational character of disaffection. Although disaffection is a process experienced by an individual, it simultaneously reflects something that is happening in interpersonal relationships. The relation of this term with other terms such as stress, anxiety, burnout or depression is studied, at the same time as the novelty it brings to the research area.
Thirdly, a characterisation of the process of disaffection was sought, indicating the starting point, and how two alternative paths open up. One alternative, the healthy one, consists of a path of creative integration that helps growth. The other alternative, the one that leads to pathology, is that generated by the process of disaffection, regarding which causes, indicators, and possible pathological outcomes can be identified. At this point we have used the studies of Fromm, justifying their relevance for this purpose. This initial characterisation will need to be completed with more theoretical and empirical studies.
Finally, we presented the operational dimensions of the concept which can be useful for the two objectives of the article: to elaborate a tool for accompaniment and to take the first step towards the creation of a psychometric scale of disaffection.

## 2 Systematic review

### 2.1 Methodology

To identify, classify, and analyse academic production addressing the phenomenon of disaffection, we conducted a systematic review which was carried out following the PRISMA 2020 Statement (Preferred Reporting Items for Systematic Reviews and Meta-analysis) available in the Supplementary material, accessible via the journal’s online submission platform (Page et al., 2021). Given the study’s aim to delimit definitional attributes and extract analytically robust conceptual components capable of supporting operationalisation, a systematic review design was selected over a scoping review, as it allowed formal methodological appraisal alongside structured evidence synthesis. For this purpose, the databases Web of Science Scopus, and PubMed were consulted, with the last search conducted on June 26, 2025. The main term in the search was disaffection, and the search strategies are detailed in the Supplementary material. After removing duplicates, the selection of records (articles) was carried out in two phases. In the first phase, titles and abstracts were screened, and all the records were required to include the term disaffection in the title. In the second phase, full texts were assessed, and articles that did not meet the Joanna Briggs Institute quality criteria (JBI) based on study type were excluded (Joana Briggs Institute, 2017).

Both authors were actively involved in the screening, data extraction, and conceptual synthesis stages. Study screening and quality appraisal were conducted independently by each author, with discrepancies discussed until consensus was reached. The first author has a background in relational psychology and has previously developed theoretical work on accompaniment and meaning, while the second author has expertise in clinical and medical research contexts. The conceptual analysis is informed by a relational and humanistic psychological framework, particularly drawing on Fromm’s work; this theoretical positioning is explicitly acknowledged in order to delimit the interpretative scope of the analysis and to enhance transparency regarding potential conceptual biases. Throughout the review and synthesis process, reflexive discussion was maintained to critically examine whether interpretative inferences were grounded in the reviewed literature rather than solely in prior theoretical commitments.

To enhance analytic validity and reliability, preliminary categorisation of findings (including the emotional, behavioural, and cognitive components, as well as the identification of underlying psychological processes) was conducted independently by both authors. The resulting classifications were compared and refined through iterative discussion until full conceptual agreement was reached. Given the interpretative and theory-informed nature of the synthesis, formal inter-rater reliability coefficients were not calculated; instead, reliability was ensured through independent coding, transparent criteria for category assignment, and consensus-based consolidation grounded in the extracted data.

### 2.2 Results

**Figure 1:**
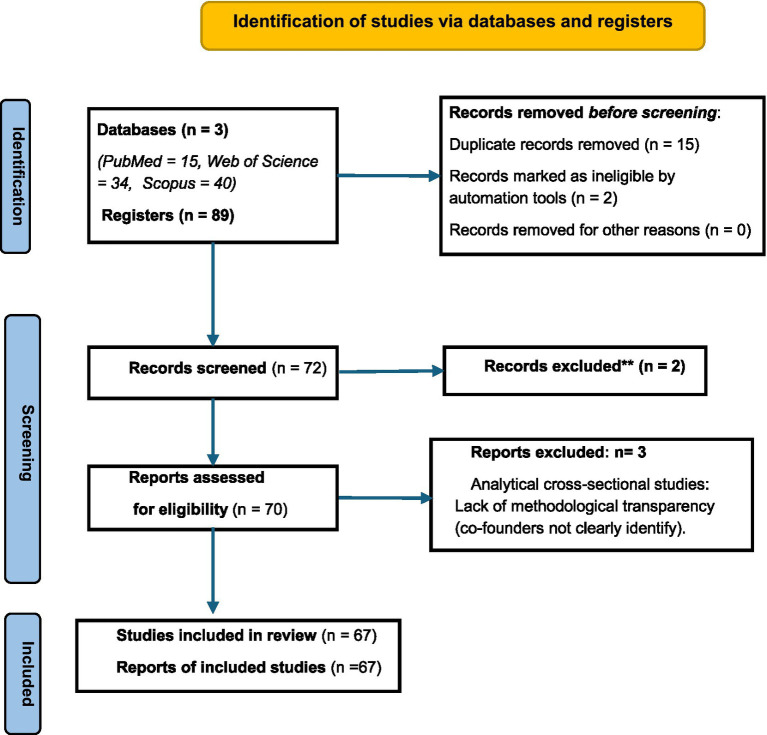
Flow search results diagram according to Page et al. (2021). No automation tools were used.

A total of 89 records were retrieved from the three databases consulted. Of these, 15 were duplicated and two were deemed ineligible by automated tools. During the initial screening, 2 records were excluded for not including the term disaffection in the title. Following the full-text assessment with the JBI critical appraisal tools, 3 additional articles were excluded, these being analytical cross-sectional studies with lack of methodological transparency (Avandeaño and Sandoval, 2016; Flowers et al., 2000; Herrington et al., 2008). Ultimately, 67 studies were included in the review. The results of the search process are presented in the PRISMA flow diagram (Figure 1).

The studies included in this review span multiple geographic regions, with a clear predominance of research conducted in Europe (*n* = 40). A smaller but significant number of studies originate from Central and South America (*n* = 12) and North America (*n* = 9). Other regions are less represented, with only a few studies conducted in Australia (*n* = 3), Asia (*n* = 1), Africa (*n* = 1) and one study with a global focus.

The thematic or social setting in which the phenomenon of disaffection is studied is diverse, with a strong concentration in the domain of politics, society, and citizens (*n* = 30), including studies such as Núñez Lira et al. (2020), Otalora (2017), Ramos (2024), and others. Education represents the second most frequent context (*n* = 24) as reflected in works by Henry and Thorsen (2020), Allan and Duckworth (2018), Poteat et al. (2025), Harber (2008), and several more. A smaller number of studies address disaffection in the field of health and professions (*n* = 6), such as Curran et al. (2015) and Garrett and Danziger (2008), while personal relationships appear as the focus in only two studies (Prus and Camara, 2010; Furrer, 2010). Lastly, religion is examined as a context for disaffection in just one study (Snape and Atkinson, 2015).

In terms of methodological design, the majority of the included studies were classified as Analytical Cross-Sectional Studies (*n* = 29) and Qualitative Research (*n* = 15), indicating a strong reliance on descriptive and interpretive approaches in the exploration of disaffection. A smaller proportion of studies fell under categories such as Text and Opinion (*n* = 8), including Expert Opinion (*n* = 4), Narrative (*n* = 2), and General Opinion (*n* = 2). Less frequently represented were Cohort Studies (*n* = 2), Quasi-Experimental Studies (*n* = 2), Cross-Sectional Studies (*n* = 1), Diagnostic Test Accuracy Studies (*n* = 1), and Systematic Reviews and Research Syntheses (*n* = 1).

The characteristics of the included studies and the quality assessment of the studies can be seen in Supplementary material.

### 2.3 Discussion

The discussion of the review findings proceeds in three steps. First, we provide a descriptive account of how disaffection has been characterised within the social sciences, organised according to the classical emotional, behavioural, and cognitive taxonomy. Second, we identify the underlying psychological processes that emerge from this literature. Finally, we synthesise these elements to offer an integrative characterisation of disaffection that prepares the ground for subsequent conceptual development.

#### 2.3.1 Social science description

The description of phenomena in the social sciences has typically followed a tripartite classification into cognitive, affective, and behavioural (Allport, 1935; Rosenberg and Hovland, 1960) which continues to be the case today (Briñol and Petty, 2006).

In general, all the articles are limited to a phenomenological description of emotions, behaviours, and understanding (cognition) of the phenomenon with superficial and descriptive analysis the psychological processes that unify and give meaning to the experience.

They describe emotions of: frustration, boredom, anxiety, sadness, irritability, and hostility without aggressiveness, emotional alienation, cynicism, demotivation, resentment, weariness or boredom, disappointment, disengagement, emotional exhaustion, apathy, lack of meaning, fatigue, absence of emotional contact, pain, and indifference.

The behaviours described are: withdrawal, avoidance of tasks or participation, academic or professional passivity, absenteeism, institutional disengagement, protest or political withdrawal, communication breakdown, work automation, evasive or impersonal behaviours, rejection of ritual practices, task abandonment, mechanical compliance without involvement, reduction of effort or performance, physical or symbolic detachment.

The understanding (cognition) of the phenomenon is focused on describing the attitudes stemming from the disappointment and the withdrawal response: distrust, detachment, sustained criticism of the system, lack of identification with the institution, scepticism towards change, perception of ineffectiveness, devaluation of the role of the other, disinterest in improvement or bonding, loss of meaning in the institution, resistance to involvement, ideological or affective ambivalence.

#### 2.3.2 Psychological processes involved in disaffection

The psychological phenomenon of disaffection emerges as a complex and gradual process, rather than as a discrete state, and is characterised by a series of interrelated emotional, cognitive, and behaviuoral transformations. Based on the detailed analysis of the literature, six core psychological processes can be described:

##### 2.3.2.1 Initial emotional engagement followed by disillusionment

Disaffection typically originates from an initial phase of engagement characterised by enthusiasm and a strong emotional investment in a project, relationship, vocation, or institutional commitment. This engagement is often rooted in personal ideals or expectations of fulfilment. However, the encounter with frustrating or dissonant realities, such as perceived institutional corruption, rigid educational demands, relational betrayals, or professional disenchantment, triggers an affective shift. Disappointment, sadness, and feelings of betrayal supplant the initial positive emotions, marking the inception of the disaffection process.

##### 2.3.2.2 Cognitive reinterpretation and progressive loss of meaning

The affective disillusionment catalyses a restructuring of cognitive appraisals. The individual begins to reinterpret the context through a progressively negative lens, generalising isolated frustrations into a more global distrust of the relationship, institution, or domain. This entails the emergence of cynical cognitions and disidentification processes whereby the individual distances their self-concept from the previously valued entity. At this stage, meaning structures become fragmented and no longer serve to orient action or sustain commitment.

##### 2.3.2.3 Behavioural withdrawal and attenuation of involvement

As cognitive and emotional processes evolve, behavioural changes ensue. These are manifested in various forms, ranging from passive withdrawal (reduced participation, apathy, indifference) to active opposition (criticism, obstruction, disengaged resistance). The observable attenuation of involvement is accompanied by a perceptible erosion of motivation and effort, signalling the progression from disaffection as an internal process to disaffection as a socially evident phenomenon.

##### 2.3.2.4 Relational erosion and identity destabilisation

Beyond disengagement from specific tasks or settings, disaffection involves the erosion of relational bonds that anchor the individual’s identity. This relational degradation is not limited to the focal object of disaffection but often generalises to broader interpersonal or institutional contexts. The person’s capacity to trust and invest relationally diminishes, leading to social withdrawal and a reduced sense of belonging, with negative repercussions on self-perception and personal agency.

##### 2.3.2.5 Failure of creative integration and polarisation towards defensive trajectories

A critical feature of disaffection is the failure to creatively integrate dissonant or frustrating experiences. Rather than adapting constructively, individuals entrenched in disaffection adopt defensive psychological strategies characterised by rigid self-protection and the suppression of vulnerability. This psychological polarisation limits flexibility, reinforces distancing behaviuors, and may foster rigid ideological stances or pervasive scepticism. Over time, this process may culminate in maladaptive patterns such as chronic cynicism or burnout.

##### 2.3.2.6 Underlying existential loss of purpose

At its deepest level, disaffection entails an existential crisis marked by the perceived loss of meaningful purpose in the relationship or project that initially served as a significant axis of orientation. This loss of purpose undermines the individual’s experiential coherence and impairs their capacity to derive satisfaction or fulfilment from engagement. The erosion of purpose thus acts as a background condition that sustains and reinforces the preceding emotional, cognitive, and behavioural changes.

##### 2.3.2.7 Conclusion of the systematic review

These six interconnected processes illuminate disaffection as a multidimensional psychological phenomenon with profound existential and relational implications. The trajectory from initial engagement to disaffection reflects not only the subjective impact of external frustrations but also internal transformations in the individual’s emotional life, cognitive frames, behaviour, relational orientation, and existential stance. Recognising and understanding these processes is critical for developing nuanced strategies of accompaniment, both preventive and therapeutic, aimed at intercepting the progression of disaffection before it culminates in pathological outcomes.

It can be seen that disaffection is a possible response by a person to a specific situation in which he or she discovers the absence of a valuable purpose or meaning where initially he or she thought that there was one.

## 3 Proposed definition, attributes of the concept and comparison with other terms

### 3.1 Proposed definition

We propose to understand disaffection as a psychological response to the loss of meaning of a purpose initially considered valuable, in which a progressive deterioration of interpersonal relationships is experienced. Unable to creatively integrate this loss, disaffection takes an adaptive self-protective form which is focused on individual satisfaction.

The meaning of the terms used here will subsequently be explained.

The process of disaffection is related to a loss of value of an initially meaningful purpose which does not necessarily involve a rupture of the interpersonal relationship and coexistence. We are not referring to the already known phenomenon in which the rupture of an affective relationship may result in the appearance of materialistic or egoistic psychological processes, compensations, superficiality of life, a search for immediate gratification, frustration and existential emptiness (Dunkel and Harbke, 2017; Kashdan and Breen, 2007; Park and Gutierrez, 2013). Indeed, in the phenomenon of disaffection, relationships can be, and often are, maintained. We are not referring to cases in which there has been an aggression and/or sentimental breakup, but rather to those in which the relationship no longer offers the meaningful purpose of value which was initially present. That which is broken is the purpose of value, and the person suffering this loss does not know how to overcome this. When going through disaffection, the person enters into adaptive, self-protective processes where he or she no longer aspires to great things, but rather merely seeks individual satisfaction. For example, the medical student who begins his or her studies with a desire to do great things and then encounters the reality in which they see the purpose of value denied, does not necessarily abandon work and relationships, but does not know how to position himself or herself creatively in the face of that situation (Rotenstein et al., 2016; Almutairi et al., 2022; Esquerda et al., 2023). As medical students advance, they face stress, distress, and a progressive loss of empathy stemming from the “hidden curriculum,” e.g., mistreatment by superiors, unrealistic expectations, and exposure to inappropriate role models (Neumann et al., 2011; Lamothe et al., 2014).

When such a loss of value of the initially meaningful purpose is experienced, the person could initiate processes of creativity and overcoming – “creative integration” – and not enter into disaffection. We will rely on Fromm to understand such creative integration in his way of understanding love. The term “integration” refers to the improvement of the interpersonal relationship and the term “creative” refers to the fact that it is not something that happens by inertia, but rather by a free intervention of the person.

Alternatively, if the person enters into disaffection, he or she starts a path that can lead to pathological processes. In order to accompany the person experiencing such a process, we seek to characterise the term disaffection and describe its causes, indicators or symptoms and pathological results. This is the only way to help the person understand how they are ascribing meaning to what they are experiencing and to be able to make decisions on how to position themselves in the face of this reality.

### 3.2 Disaffection, an individual and relational process

We propose to understand that disaffection is simultaneously an individual and relational process. Although the disaffected person is one, we believe that the experience of disaffection is relational from its root, i.e., that which happens within oneself, is a reflection of what is happening in the relationship.

This position is in line with an axiom of anthropological nature: what is relational in the person does not seem to be something accidental, but constitutive of who one is (A review of this proposal can be found in Akrivou et al., 2018 and its neuroscientific references. Luis et al., 2022). This anthropological presupposition need not be shared by everyone, but pointing it out gives transparency to the options we present and at the same time indicates their scope.

The understanding of disaffection as a process that is both individual and relational is arrived at by the following argumentation, which is stated here and then developed and justified (Orón Semper, 2019):

1) To understand the stress experienced by a person, it is more important to understand how the person ascribes meaning to the event than the event itself.
2) The process of ascribing meaning to an event is always an interpersonal social issue because events are understood by the way they affect interpersonal relationships.
3) The quality of the interpersonal relationship is the major reference used by the person in his or her evaluations, since this quality is the major predictor of both the success and the life expectancy of the person.

To this end, we point out below how the purpose of meaning in life – what is considered valuable by the person – and the process of ascribing meaning to reality are essentially relational (Orón Semper, 2023).

That the person needs orientation, and orients his or her life, following a purpose of meaning has been well developed (among others) by Frankl, the relevance of whose work is evident, for example, in explaining the situation of the student and the teacher (Cachón Zagalaz, 2021; Frankl, 1959; Gabrys and Langdale, 2011). In Frankl’s proposal for the search for meaning, he articulates the relations between work, interpersonal relationships, and suffering. Given Frankl’s transcendental anthropology (Selles, 2015), all these relations end up pivoting on the interpersonal relationships.

The stress commonly associated with the experience of disaffection is also relational since it is not possible to understand the stress experienced without the meaning being ascribed by the person to the event, this meaning being intrinsically relational. Studies on stress show that, to understand its existence, one must focus more on the understanding and significance that the person makes of the event, rather than on the event itself (Boals, 2018; Espinoza Ortíz et al., 2018; García et al., 2018; Lería Dulčić and Salgado Roa, 2016; Pacheco, 2021).

But reality is not experienced in isolation; rather, its meaning is constructed within the framework of interpersonal relationships. Saaty (2023) shows how the expression of feelings in personal narratives is influenced by the social environment. Dong and Li (2024) show that the perception of work stress depends on psychosocial support rather than on the event itself. García-Santos et al. (2024) emphasise that a sense of purpose arises from interaction with others. Leal Riquelme and Herrera Guerrero (2009) also assert that the personal act only acquires meaning within an intersubjective structure.

In addition, it is known that the quality of interpersonal relationships is the factor most valued by the person, this quality being the greatest predictor of both the success and death of the person (Orón Semper, 2019: 19). It therefore makes sense to connect the loss of a purpose of value with the deterioration of the quality of interpersonal relationships, since this quality is the fundamental purpose of value for the person.

This does not negate the individual dimension of disaffection given that the process of ascribing meaning, the search for meaning and the experience of stress all occur within the person whilst simultaneously occurring within the context of the interpersonal relationship.

### 3.3 Comparison with other terms

When a specific term is proposed, it must be shown that it has its own semantic field, distinct from other similar terms. Other terms such as crisis, stress, burnout, anxiety or depression have different semantic fields. Crisis, referring to a person’s psychological experience, indicates that certain ways of understanding reality no longer serve to position oneself accordingly and can be part of a perfectly healthy process such as the formation of one’s own identity (Erikson, 1968; Marcia, 1980; Marcia et al., 2012). Stress refers to the tension experienced by the person and can be divided into three categories. It can be normative when it is sought for the healthy development of the person; it can be tolerable, when it is not sought but presents an opportunity for personal growth; and it can be toxic, when stress is experienced in a harmful way. The dividing line between the tolerable and the toxic is not easy to recognise *a priori* (Lupien et al., 2018; McEwen, 2017; Shonkoff et al., 2012). Burnout is not a disease, a diagnosis or a syndrome, but rather a recognition on the part of the person of having reached their limit, acknowledging feeling broken, exhausted, depleted, unresourced, and disconnected (Bianchi et al., 2022; Hillert et al., 2020; Salvagioni et al., 2017). Anxiety and depression share a negative affect base, which explains their high co-morbidity. Nonetheless, they present key differences in their manifestation. While anxiety is characterised by hypervigilance and anticipatory response to danger, depression is associated with anhedonia and hopelessness, affecting motivation and pleasure. Furthermore, in neurobiological terms, anxiety is related to hyperactivity in the amygdala, while depression is more related to the prefrontal and reward system. However, both situations share symptoms such as fatigue, sleep disturbances, and difficulties in concentration, which indicates an important area of overlap between them (Eysenck and Fajkowska, 2022; Gaspersz et al., 2023; Shaw and Starr, 2024).

The terms crisis, stress, burnout, anxiety or depression refer to what a subject experiences in a descriptive way, without referring to a specific cause or process, whereas disaffection refers to a specific process, which, depending on how it is experienced, may or may not lead to crisis, stress, burnout, anxiety or depression. Disaffection refers to the response that appears in a specific context, which allows us to identify the psychological processes to accompany with more precision and specificity. Moreover, disaffection can occur without an obvious manifestation and can be easily concealed by a person with social and personal management resources. In such a case, crises, stress, burnout, anxiety or depression would not initially appear, but a need for accompaniment could be detected.

## 4 Toward an understanding of the process of disaffection

### 4.1 Appropriateness of Erich Fromm’s studies for an initial characterisation of disaffection

The theoretical discussion grounded in Fromm’s work does not precede the empirical analysis but serves as an interpretative framework through which the psychological processes identified in the systematic review can be structurally organised and further articulated.

To be able to provide accompaniment, it is necessary to go beyond the phenomenological description and to know the psychological processes so that the person can understand his or her personal dynamics and make decisions. To characterise the psychological processes of disaffection we have turned to the works of the psychoanalyst Fromm, given that he carried out a deep investigation into the rupture of primary bonds, showing that, after this rupture, the person can follow two different paths. On one path, the healthy one, the person grows in a free act, which improves their way of relating to the experience of love, in such a way that the person affirms his or her individuality and freedom by affirming the individuality and freedom of the other. On the other path, the unhealthy one, the person goes through psychological processes that lead to pathological situations consisting of a false affirmation of individuality which Fromm calls secondary bonds. The way Fromm understands love enables us to unify (within the person) the processes of individualisation and the relational processes.

Although Fromm does not use the term disaffection and the scope of his work is much broader, this does not negate the term’s usefulness in this context. Disaffection can be understood as a passage from primary to secondary bonds, which does not mean that every such transition is exclusively due to disaffection. The description of this transition is a good starting point for understanding disaffection because the deterioration of bonds is because there is no longer a meaningful purpose of value, which for Fromm would be “to work and to love”.

Love and work “affirm the individuality of the self and at the same time unite the individual with others and nature” (Fromm, 1972:299). “His spontaneous activity, work and love, capable of re-uniting him with the world, no longer by means of primary bonds, but by saving his character as a free and independent individual” (Fromm, 1972: 62) Love is not a mere affective state, but the process of personal affirmation that results from affirming the individuality of another (Fromm, 1972: 146–148). Working is what we might call humanising the world in the sense of transforming it into a resource for love (Fromm, 1972: 198–199). The effects of love and work are an increase in individuation and integration (Fromm, 1972: 62, 172, 299). The sequence of breaking bonds – the person either growing in love and work, or regressing by establishing secondary bonds – is constantly repeated throughout life. Each increase in freedom and individuation again produces the dilemma of whether to enter into love and work or processes of arrest or regression. “Man can only choose between two possibilities: regression or progression.” “There is only the regressive and the progressive solution” (Fromm, 1982:65). There is no neutrality possible, stagnation is already pathological (Fromm, 1972, 189–190).

After the evidence that the primary bonds need to be reinterpreted, the person can move in a creative and a free way towards work and love, or towards secondary bonds, which are false forms of affirming individuality, and thus pathological.

### 4.2 Causes, indicators and pathological outflow according to Erich Fromm

To enhance analytical clarity and to facilitate the systematic identification of the elements that may later inform conceptual operationalisation, the contributions derived from Fromm’s framework are presented below in a structured format. For Fromm, the annulment of the person’s interiority is a common factor in all the educational beliefs and practices that favour the transition towards pathological bonds. This annulment leads to the person placing himself or herself alone in the face of external demands. These educational issues are:

1) Understanding the educational process as disapproval and annulment of the original thought results in killing the person’s curiosity and inner desires to know the truth (Fromm, 1972: 283).
2) Threats and punishments eliminate the child’s originality, his or her inner self, and “the child starts with giving up the expression of his feelings, and eventually gives up the very feeling itself” (Fromm, 1972: 278–279).
3) Educating in certain fictitious ideals (Fromm, 1972: 303) permits a rationalisation of the different actions to build an idealism which justifies the degradation by dressing it up as something great (Fromm, 1972: 269, 272).
4) Focusing on introducing data by rote learning, i. e, teaching data disconnected from any meaning and relationship with life.
5) Educating in a positivist perspective, in which only that which is empirically demonstrable exists. This dissociates reality (which is associated with something external and given) and the subject’s intention, and leads to the interior being regarded as an issue exclusive to the subject, thus giving rise to relativism and absurdity. For Fromm, the healthy search for truth is since that the individual having interests that arise from his process of individuation. When the educational interest is separated from the individuation process, the stimulus to search for truth disappears – everything is mere data to be stored.
6) Learning everything as if it were extremely simple, which goes against the complexity of life itself. This perspective assumes a very reductive cause-and-effect mechanism. According to Fromm, it favours scepticism, cynicism, a childish way of accepting authority, and irresponsibility.
7) Seeking no order and therefore serving no end. The term order does not refer to an organisation of things, but to the fact that the means are “ordered” in the service of the ends. When order disappears, one lives superficially without reflection, looking for satisfaction without knowing the origin of one’s desires.
8) Accepting instrumentalisation as the means of relating to reality. Everything becomes an instrument. Even oneself is an instrument, one more cog in the wheel, which results in a loss of identity. This loss of identity leads to “conformism.”
9) Following desires without knowing their origin. One simply desires and follows one’s desires without knowing how they are organised or integrated with other aspects of the person. Desires, even if they are one’s own, are experienced as explosions unrelated to other processes of the person.
10) Idolising one’s successes, which are understood as achievements connected with idealised images rather than with the experience of love and work.

Items 4 to 10 are listed in (Fromm, 1972: 284–294).

This educational environment does not necessarily lead to the person forming secondary bonds, given that the person could, despite this environment, creatively place himself or herself by loving and working. However, if this is not the case, the person moves towards secondary bonds. There are some symptoms which indicate that the person is moving in a pathological direction:

1) Loss of meaning of life (Fromm, 1972: 314, 1955: 61–64, 85, 1947: 11, 145).
2) Non-formation or deterioration of personal identity (Fromm, 1972: 188–189, 220, 240, 291, 1955: 59–61).
3) Deterioration of the quality of personal relationships and lack of empathy coupled with a fall into symbiosis of mutual dependence (Fromm, 1972: 193, 213; 1982: 55–61, 1955: 152).
4) Search for security, possession and control. The person wants to be someone because of what they have rather than who they are (Fromm, 1972: 55, 206, 1982: 13–18).
5) Moral disengagement resulting from rationalisations in which apparent reason and the appearance of love are used to justify decisions, combined with a lack of responsibility concerning the effects of one’s actions on others (Fromm, 1982: 22, 70–73; 1955: 61–63; 1972: 224, 229, 266).
6) Compensatory mechanisms to alleviate frustration when the person does not receive the required help from the “magic helpers” (that coincide with secondary bonds) (Fromm, 1972: 211, 212).
7) The emergence of a new motivational system: utilitarian, individualistic, short-term, fixed, materialistic, non-integrated and so on.

And when a person reaches pathology, we discover different ways of falsely affirming individuality through secondary bonds. Although the person is always oriented towards transcendence, these are false ways of achieving this. Healthy transcendence is love and work by which one affirms oneself by affirming the other. The unhealthy ways of transcending oneself in search of individuality are to do so in the service of some things:

1) *Oedipus complex*. One wants to go back to where one came from, i.e., going back to the mother. Fromm distances himself from Freud in that he does not regard this complex as having sexual connotations (Fromm, 1972: 211, 1982, 50–54). One characterises the aspirations of one’s parents, in particular the mother, and seeks to adjust to and satisfy such aspirations in an attempt to maintain the initial healthy bond, but now, in an unhealthy way, with a secondary pathological bond.
2) Selfishness. While selfishness might seem to stem from a high valuation of oneself, in reality this stems from a self-dislike which leads to a need to adorn oneself with things. One can also fall into a servitude to one’s successes (Fromm, 1947: 22, 1972: 147–149). The person feels of value due to the possession of things and as such, a pathological individuation is reached.
3) Narcissism. Similar to selfishness, there is an underlying self-dislikeness that one seeks to overcome through a constant anxiety concerning one’s image and how one is perceived by Fromm (1972: 148; 1982: 32–49, 1955: 28–34). The interiority of the person is thus annulled and the person is reduced to a series of characteristics (idealised and/or imagined) that the person seeks to achieve and/or maintain.
4) Masochism. This addresses the need to transcend oneself by annulling one’s individuality through submission to another – be it a person or an institution –that gives apparent weight and value to oneself. It does not necessarily involve causing harm to oneself, although this may occur in more extreme cases (Fromm, 1955: 28–34, 1972: chap V). One’s individuality is sought by associating with and placing oneself under the influence of another positively valued individuality (personal or institutional).
5) Sadism. This appears when one believes to find the solution to the individuality that one does not achieve healthily through control over others. It does not have to involve causing pain to another, sometimes taking an almost opposite form, such as putting the other in a “golden cage” for the exercise of power (Fromm, 1955: 28–34, 1972: chap V). One is what one is under who or what one succeeds in subduing or controlling.
6) Destructiveness. The person is a kind of non-revolutionary rebel who seeks to be different without being original. This way of solving the conflict consists of affirming oneself by a no rather than by a yes. It is a movement contrary to love. The person also seeks to transcend him or herself by annulling that which it cannot love (Fromm 1982: 12–13, 1955: 33–35: 2022).

Fromm states that all these negative ways of resolving individuation have initial forms that are not pathological, but simply generate a disruption that has to be attended to. In other words, a transit from health to illness is generated (Fromm, 1982: 15, 22, 40, 46, 54, 56, 61). This idea of “transit” is very important to understand how the term disaffection participates in both healthy and pathological dynamics.

### 4.3 Dimensions of the concept of disaffection for accompaniment

For the work to be useful for accompaniment in cases of disaffection, a phenomenological description is not enough, but it is necessary to offer the person clues as to what personal processes (or causes) lead him or her to initiate a process of disaffection, and how to recognise its intensity (indicators or symptoms) and what the pathological outflows are, which may have an initial social approval. To this end, to facilitate accompaniment in disaffection, we can rewrite Fromm’s contributions in a way that better suits our purposes. Thus, instead of speaking of causes, indicators, and pathologies, we will refer to beliefs, attitudes, and pathologies.

The causes of disaffection do not necessarily operate in the subject; it is the person who can either initiate processes of love and work, or internalise the principles of the educational environment which fosters disaffection and initiate a process leading to secondary bonds. In the latter case, it is not simply a question of a person experiencing an educational environment which favours disaffection, but rather that the person has internalised the underlying principles of this educational environment. Because these principles need to be adopted in some way to operate, it makes sense to reformulate them as beliefs rather than causes. In addition, re-understanding causes as beliefs makes it possible to identify whether or not the process of disaffection is taking place in a specific subject.

Understanding causes as beliefs also aids accompaniment. Discovering the extent and intensity of these beliefs in a person not only makes it possible to know the risk of him or her going through the process of disaffection, but also allows accompaniment to focus on studying how and why these beliefs were generated and how they can be re-signified by healing relationships and having new experiences. The common denominator of the different beliefs which we list here is that they isolate the person from his or her interiority and meaningful personal relationships. In addition, each belief explicitly or implicitly entails a specific affirmation and a specific negation:

1) I believe that life is about responding to what is expected of one, with no need for initiative or purpose beyond meeting external demands.
2) I believe that decisions should be made based on the benefits or otherwise that they may bring, given that what matters is to avoid punishment and seek reward.
3) I believe that learning means retaining the information needed to make things work or to succeed, without any need to question its meaning or purpose.
4) I believe that only that which is empirically demonstrable is objective and true; the subjective and individual intentions belong to the private sphere and are not relevant to knowing reality.
5) I believe that life has no specific meaning, but is merely a chain of causes and effects.
6) I believe that events happen in temporal order without any deep connection between them; life is simply a sequence of events governed by the agenda and the calendar and not by the realisation of a purpose of value.
7) I believe that relationships with others have value to the extent that they are useful or effective in achieving individual goals that one has set for oneself.
8) I believe that thoughts, desires, and feelings arise spontaneously in one, with an uncertain origin. The important thing is to act following these thoughts, desires, and feelings without needing to know their origin.
9) I believe that success – understood as the achievement of the goals one sets – is the key to peace of mind and satisfaction in life.
10) I believe that it is best to organise one’s life following one’s own tastes, with how one thinks that things should be or with an ideal version of a successful life, as opposed to being open to the surprise and novelty of the people one meets.

As with causes and beliefs, the indicators of disaffection can be usefully reformulated as existential attitudes or ways of facing reality. Formulating them as existential attitudes makes it possible to discover to what extent the person has already organised his or her life according to the beliefs (causes) of disaffection and can enable a more objective assessment of the person’s situation, which is crucial to accompaniment. The common denominator of these attitudes is that the person experiences mere functional adaptive processes, taking their well-being as a reference.

1) In my daily life, I do not experience a clear orientation. I neither project myself beyond day-to-day affairs nor do I articulate a meaning that transcends these.
2) In my daily life, my identity is diluted in the multiplicity of tasks and commitments; I adapt to each environment without questioning my place in it.
3) In my daily life, I perceive that my interpersonal relationships lack depth and do not generate authentic satisfaction.
4) In my daily life, I recurrently focus on how to control situations and ensure that my goals are achieved.
5) In my daily life, I justify my actions to avoid questioning what is happening to me and thus, escape responsibility for my decisions.
6) In my daily life, I relate to others by how situations affect me, without considering what the other person is going through.
7) In my daily life, my usual way of dealing with suffering consists of seeking immediate compensation through pleasure (food, sex, shopping or other gratifications).
8) In my daily life, my motivations tend to be marked by materialism, individualism and short-term orientation.

To identify pathological manifestations, it is useful to recognise false forms of affirming individuality. Given that such forms can lead to situations which become problems in themselves, recognising them is helpful for accompaniment. Furthermore, these forms are not easily identified because they may initially be socially accepted:

1) Return to the familiar: The person seeks security in familiar patterns or childhood experiences, such as those related to the Oedipus complex. The individual believes to be finding their individuality in the connection with their origin, but the person is diluting their uniqueness by adopting characteristics or behaviours that imitate their parents. The person thus nullifies their ability to define a self of their own.
2) Hoarding: In this manifestation, the individual confuses their identity with what they own. A false appearance of individuality is constructed by the accumulation of material objects, in the belief that having determines being. However, this strategy detaches the self from its essence, reducing it to a superficial image.
3) Self-absorption: Here, the person identifies themselves with certain individual characteristics that they consider valuable or perfect, such as physical, intellectual or emotional attributes. This narcissism generates a false perception of uniqueness by reducing identity to a set of specific traits, disconnected from the complexity and depth that define the authentic self.
4) Subordination to others: In this case, the individual seeks to assert their uniqueness and sense of self through submission to other people or institutions. Similar to masochism, they mistakenly perceive the individuality of the one who dominates them as the guarantor of their own identity.
5) Subordination of others: The person attempts to assert their individuality and sense of self by exercising control or domination over others. Similarly to sadism, they mistakenly believe that the ability to subjugate others validates their uniqueness and individuality.
6) Destructiveness: The individual finds a false affirmation of the self in the denial or destruction of that which they cannot either integrate into their identity or understand, which can include themselves.

### 4.4 Limitations and future research directions

Several limitations should be acknowledged. Although the systematic review followed PRISMA 2020 guidelines and included critical appraisal of study quality, the proposed conceptualisation of disaffection has not yet undergone empirical validation. As is characteristic of theory-driven and concept-oriented reviews, the contribution lies primarily in analytical clarification rather than empirical testing (Page et al., 2021). Accordingly, its predictive capacity and discriminant validity remain to be examined.

The conceptual synthesis is predominantly grounded in Erich Fromm’s theoretical framework. While this provides depth and coherence in understanding disaffection as an individual and relational process, it also circumscribes the interpretative scope. This anchoring represents a deliberate and transparent positioning rather than an exhaustive integration of psychological perspectives. Future work may benefit from incorporating complementary approaches within psychoanalytic traditions, clinical and developmental psychology, sociology, neuropsychology, and social psychology, thereby refining or extending the present model.

As with any systematic review, the results are contingent upon the quality, distribution, and conceptual consistency of the available literature. Despite rigorous inclusion criteria and methodological appraisal, relevant dimensions of disaffection may remain underrepresented due to disciplinary fragmentation or publication patterns, a recognised limitation in systematic syntheses of complex psychosocial phenomena (Munn et al., 2018).

Building on these considerations, empirical research is required to test the conceptual specificity of disaffection and its discriminant validity in relation to constructs such as burnout, anxiety, depression, demotivation, and life crisis. The development and validation of reliable psychometric instruments and diagnostic scales would enable identification of different stages and manifestations across populations and relational contexts, facilitating early detection and preventive intervention.

Longitudinal designs may clarify developmental trajectories and examine whether disaffection functions as a transitional process between well-being, stress, and psychopathological outcomes. Experimental and applied studies are also needed to evaluate evidence-based interventions capable of mitigating or reversing disaffection processes in educational, clinical, and organisational settings. Integration of the construct into professional training programs and interdisciplinary research will further contribute to consolidating its academic and practical relevance.

Finally, given the centrality of relational space in accompaniment practices, the growing incorporation of artificial intelligence in clinical and psychological contexts warrants careful scrutiny. While AI may serve as a supportive tool, its uncritical use risks reinforcing instrumentalisation unless embedded within explicitly human-centered relational frameworks (Lidströmer et al., 2022a,b). Future research should therefore examine the conditions under which technological mediation enhances or undermines relational depth in addressing disaffection.

## 5 Conclusion

The concept of disaffection presented here seeks to contribute to personal accompaniment by offering a specific theoretical construct applicable across multiple fields. Unlike terms such as demotivation, burnout, anxiety, and depression, which operate within broader and less specific conceptual contexts, disaffection is defined as a psychological response of self-protection in the face of a progressive loss of value of an initially meaningful purpose. Since the value of this purpose is embedded within relational contexts, its erosion entails either the rupture of bonds or their emptying of transcendent significance. This definition allows for a more precise and theoretically grounded intervention framework.

The proposed tripartite structure (causes, indicators, and pathological outflows) provides a coherent schema for early detection and progressive accompaniment. By reformulating causes as dysfunctional beliefs, the model facilitates both preventive and corrective cognitive work, enabling intervention during incipient phases before external manifestations become consolidated.

Disaffection is conceptualised as a simultaneously individual and relational process. The relational dimension, illuminated through Fromm’s contributions, expands the scope of intervention beyond intrapsychic work to include the qualitative transformation of bonds. This perspective makes it possible to address group and institutional relational dynamics that may either foster or mitigate disaffection.

The systematisation of beliefs (causes), existential attitudes (indicators), and false assertions of individuality provides a structured framework for identifying different phases of the process, promoting earlier action, and encouraging creative responses before pathological manifestations arise. Together, these elements offer an articulated conceptual foundation for understanding and accompanying disaffection as a distinct psychological and relational phenomenon.

## Data Availability

The original contributions presented in the study are included in the article/Supplementary material, further inquiries can be directed to the corresponding author/s.

## References

[ref1] Ahmad R. Nejati M. Farr-Wharton B. (2024). Impact of leadership on unethical pro-organizational behavior: a systematic literature review. J. Leadership Organ. Stud. 31, 45–60. doi: 10.1177/15480518241265399

[ref2] Akrivou K. Orón Semper J. V. Scalzo G. (2018). The inter processual Self: Towards a Personalist Virtue Ethics Proposal for human Agency. Newcastle upon Tyne: Cambridge Scholars Publishing.

[ref3] Allan D. Duckworth V. (2018). Voices of disaffection: disengaged and disruptive youths or agents of change and self-empowerment? Br. J. Spec. Educ. 45, 43–60. doi: 10.1111/1467-8578.12201

[ref4] Allport G. W. (1935). “Attitudes,” in Handbook of Social Psychology, ed. Murchison C. (Worcester, MA: Clark University Press), 798–844.

[ref5] Almutairi A. F. Salam M. Alhajri N. Alturki N. Almansour R. A. Almutairi B. S. (2022). Prevalence of burnout in medical students: a systematic review and meta-analysis. Int. J. Soc. Psychiatry 68, 1157–1170. doi: 10.1177/00207640221106691, 35775726

[ref6] Asensio S. Orón J.V. Senabre I. Pardo J. (2023). Afabilidad en el tesón, energía y motivación del alumno. In López González J. Martín Martínez L. (eds.) Educar a través del acompañamiento y la relación. Volumen 1 (43–59). Barcelona: Octaedro. 978–84–19690-54-8

[ref7] Avandeaño O. Sandoval P. (2016). Political disaffection and stability of the election results in Chile, 1993–2009. Perfiles Latinoam. 24, 175–198. doi: 10.18504/pl2447-010-2016

[ref8] Bianchi R. Schonfeld I. S. Laurent E. (2022). Should burnout be conceptualized as a mental disorder? Behav. Sci. 12:82. doi: 10.3390/bs12030082, 35323401 PMC8945132

[ref9] Boals A. (2018). Trauma in the eye of the beholder: objective and subjective definitions of trauma. J. Psychother. Integr. 28, 75–89. doi: 10.1037/int0000092

[ref10] Boswell W. R. Boudreau J. W. Tichy J. (2005). The relationship between employee job change and job satisfaction: the honeymoon-hangover effect. J. Appl. Psychol. 90, 882–892. doi: 10.1037/0021-9010.90.5.882, 16162061

[ref11] Briñol P. Petty R. E. (2006). “Fundamentos de Psicología Social,” in Psicología Social, eds. Morales J. F. Huici C. Moya M. Gaviria E. (Madrid: McGraw-Hill), 459–486.

[ref12] Cachón Zagalaz J. (2021). *Compromiso con la carrera docente, frustración de las necesidades psicológicas y resiliencia personal del futuro profesorado de Infantil, Primaria y Secundaria*. [doctoral thesis]. Granada: Universidad de Granada.

[ref13] Clarke N. Jennings W. Moss J. Stoker G. (2018). The good Politician: Folk Theories, Political Interaction, and the rise of anti-Politics. Oxford: Oxford University Press.

[ref14] Curran T. Hill A. P. Appleton P. R. Vallerand R. J. Standage M. (2015). The psychology of passion: a meta-analytical review of a decade of research on intrapersonal outcomes. Motiv. Emot. 39, 631–655. doi: 10.1007/s11031-015-9503-0

[ref15] Díaz A. J. Pérez Cano D. Ramos R. Yanes F. Delgado J. M. S. (2010). Factores psicosociales motivacionales y estado de salud. Med. Segur. Trab. 56, 3–16. doi: 10.4321/S0465-546X2010000100002

[ref16] Dong R. K. Li X. (2024). Psychological safety and psychosocial safety climate in the workplace: a bibliometric analysis and systematic review towards a research agenda. J. Saf. Res. 88, 35–50. doi: 10.1016/j.jsr.2024.08.001 39998511

[ref17] Dunkel C. S. Harbke C. R. (2017). Attachment, self-esteem, and materialistic values following relationship dissolution. Pers. Individ. Dif. 114, 64–69. doi: 10.1016/j.paid.2017.03.029

[ref18] Erikson E. H. (1968). Identity: Youth and Crisis. New York: W.W. Norton & Company.

[ref19] Espinoza Ortíz A. A. Pernas Álvarez I. A. González Maldonado R. L. (2018). Consideraciones teórico metodológicas y prácticas acerca del estrés. Human. Méd. 18, 697–711. Available online at: https://humanidadesmedicas.sld.cu/index.php/hm/article/view/1264

[ref102] Esquerda M. Yuguero O. Viñas J. Pifarré J. (2023). Academic climate and psychopathological symptomatology in Spanish medical students. BMC Medical Education, 23, 45. doi: 10.1186/s12909-023-04009-2 37936105 PMC10631074

[ref20] Eysenck M. W. Fajkowska M. (2022). Anxiety and depression: a systematic review of cognitive and affective dimensions. Int. J. Clin. Health Psychol. 22:7191. doi: 10.1080/02699931.2017.1330255

[ref21] Flowers C. Robinson B. E. Carroll J. J. (2000). Criterion-related validity of the marital disaffection scale as a measure of marital estrangement. Psychol. Rep. 86, 1101–1103. doi: 10.2466/pr0.2000.86.3c.1101, 10932563

[ref22] Frankl V. E. (1959). Man’s search for Meaning. Boston: Beacon Press.

[ref23] Fromm E. (1947). Man for himself: An Inquiry into the Psychology of Ethics. New York: Rinehart & Company.

[ref24] Fromm E. (1955). The sane Society. London: Routledge.

[ref25] Fromm E. (1972). El miedo a la libertad. (versión castellana de Germani Gino). Buenos Aires: Paidos.

[ref26] Fromm E. (1982). El corazón del hombre. Su potencia para el bien o para el mal. Ciudad Autónoma de Buenos Aires: Fondo de Cultura Económica.

[ref27] Fromm E. (2022). Anatomía de la destructividad humana. 1st Edn. Madrid: Siglo XXI de España Editores, S.A.

[ref103] Fulmer C. B. Sinclair R. R. (2023). Burnout Among Pastors in Relation to Congregation Member and Church Organizational Outcomes. Review of Religious Research, 65, 62–90. doi: 10.1177/0034673X231176075

[ref28] Furrer C. J. (2010). Capturing the friendship context with a collective property: friendship group engagement vs. disaffection. J. Adolesc. 33, 853–867. doi: 10.1016/j.adolescence.2010.07.003, 20732710

[ref101] Gabrys B. J. Langdale J. A. (2011). How to Succeed as a Scientist: From Postdoc to Professor. Cambridge: Cambridge University Press.

[ref29] García F. E. Vega Rojas N. Briones Araya F. (2018). Rumiación, crecimiento y sintomatología postraumática en personas que han vivido experiencias altamente estresantes. Av. Psicol. Latinoam. 36, 443–457. doi: 10.12804/revistas.urosario.edu.co/apl/a.4983

[ref30] García-Santos C. Florez-Jiménez M. P. Lleó-de-Nalda A. (2024). Purpose Trends Report [Informe]. Navarra: Universidad de Navarra.

[ref31] Garrett R. K. Danziger J. N. (2008). Disaffection or expected outcomes: understanding personal internet use during work. J. Comput.-Mediat. Commun. 13, 937–958. doi: 10.1111/j.1083-6101.2008.00425.x

[ref32] Gaspersz R. Lamers F. Kent J. M. Beekman A. T. F. Smit J. H. Penninx B. W. J. H. (2023). The dimensional structure of anxiety and depression: a meta-analysis of factor-analytic studies. J. Affect. Disord. 320, 419–435. doi: 10.1016/j.jad.2023.05.012

[ref33] Harber C. (2008). Perpetrating disaffection: schooling as an international problem. Educ. Stud. 34, 457–467. doi: 10.1080/03055690802288445

[ref34] Henry A. Thorsen C. (2020). Disaffection and agentic engagement: ‘redesigning’ activities to enable authentic self-expression. Lang. Teach. Res. 24, 456–475. doi: 10.1177/1362168818795976

[ref35] Heo Y. Kim K. J. Kim H. (2020). From emotional exhaustion to cynicism in academic burnout among Korean high school students: focusing on the mediation effects of hatred of academic work. Stress. Health 36, 376–383. doi: 10.1002/smi.2936 32073215

[ref36] Herrington R. L. Mitchell A. E. Castellani A. M. Joseph J. I. Snyder D. K. Gleaves D. H. (2008). Assessing disharmony and disaffection in intimate relationships: revision of the marital satisfaction inventory factor scales. Psychol. Assess. 20, 341–350. doi: 10.1037/a0013759, 19086757

[ref37] Higgins J. P. T. (2019). Cochrane Handbook for Systematic Reviews of Interventions. 2nd Edn. Hoboken, NJ: John Wiley & Sons.

[ref38] Hillert A. Albrecht A. Voderholzer U. (2020). The burnout phenomenon: a résumé after more than 15,000 scientific publications. Front. Psych. 11:519237. doi: 10.3389/fpsyt.2020.519237, 33424648 PMC7793987

[ref39] Jennings W. Stoker G. Twyman J. (2016). The dimensions and impact of political discontent in Britain. Parliament. Aff. 69, 876–900. doi: 10.1093/pa/gsv065

[ref40] Joana Briggs Institute. (2017). Available online at: https://jbi.global/critical-appraisal-tools (Accessed July 29, 2020)

[ref41] Kashdan T. B. Breen W. E. (2007). Materialism and experiential avoidance as predictors of life meaning following romantic relationship dissolution. J. Posit. Psychol. 2, 240–252. doi: 10.1080/17439760701552345

[ref42] Lamothe M. Boujut E. Zenasni F. Sultan S. (2014). Empathy in medical students: a systematic review. Med. Teach. 36, 635–652. doi: 10.3109/0142159X.2014.909587

[ref87] Lavner J. A. Karney B. R. Williamson H. C. Bradbury T. N. (2018). Workload and marital satisfaction over time: Testing lagged spillover and crossover effects during the newlywed years. Journal of Vocational Behavior, 107, 336–347. doi: 10.1016/j.jvb.2017.05.002 PMC5658017 29081533

[ref43] Le Bretón D. (2023). Desaparecer de sí. Una tentación contemporánea. 5ª Edn Biblioteca de ensayo 86 (serie mayor) Madrid: Siruela.

[ref44] Leal Riquelme R. Herrera Guerrero B. (2009). La constitución de significado en el ámbito de las relaciones intersubjetivas: El acto personal y la acción social. Alpha 28, 135–151. doi: 10.4067/S0718-22012009000100009

[ref45] Lee M. Cho S. (2019). A multilevel analysis of change in hatred of academic work during high school: focusing on the sociocultural background of Korea. Learn. Individ. Differ. 74:101755. doi: 10.1017/jgc.2019.6

[ref46] Lee M. Lee K.-J. Lee S. M. Cho S. (2020). From emotional exhaustion to cynicism in academic burnout among Korean high school students: focusing on the mediation effects of hatred of academic work. Stress. Health 36, 376–383. doi: 10.1002/smi.2936, 32073215

[ref47] Lería Dulčić F. J. Salgado Roa J. (2016). Estrés postraumático y estrés subjetivo en estudiantes universitarios tras aluvión de barro. Cienc. Psicol. 10, 163–173. doi: 10.22235/cp.v10i2.1250

[ref48] Li C. Ivarsson A. Stenling A. (2022). Relationship between athlete stress and burnout: a systematic review and meta-analysis. Psychol. Sport Exerc. 58:102077. doi: 10.1080/1612197X.2021.1987503

[ref49] Lidströmer N. Aresu F. Ashrafian H. (2022a). “Introductory approaches for applying artificial intelligence in clinical medicine,” in Artificial Intelligence in Medicine, eds. Lidströmer N. Ashrafian H. (Cham: Springer). doi: 10.1007/978-3-030-64573-1_18

[ref50] Lidströmer N. Aresu F. Ashrafian H. (2022b). “Basic concepts of artificial intelligence: primed for clinicians,” in Artificial Intelligence in Medicine, eds. Lidströmer N. Ashrafian H. (Cham: Springer).

[ref51] Linden M. (2011). “Posttraumatic embitterment disorder (PTED),” in Embitterment: Societal, Psychological, and Clinical Perspectives, eds. Linden M. Maercker A. (Heidelberg: Springer), 47–64.

[ref52] Linden M. Baumann K. Rotter M. Schippan B. (2008). Diagnostic criteria and the standardized diagnostic interview for posttraumatic embitterment disorder (PTED). Int. J. Soc. Psychiatry 12, 93–96. doi: 10.1080/13651500701580478, 24916618

[ref53] Linden M. Muschalla B. (2011). “Embitterment and the workplace,” in Embitterment: Societal, Psychological, and Clinical Perspectives, eds. Linden M. Maercker A. (Heidelberg: Springer), 127–138.

[ref54] Luis E. O. Akrivou K. Bermejo-Martins E. Scalzo G. Orón J. V. (2022). The interprocessual-self theory in support of human neuroscience studies. Front. Psychol. 12:686928. doi: 10.3389/fpsyg.2021.686928, 35153881 PMC8832125

[ref55] Lupien S. J. Juster R. P. Raymond C. Marin M. F. (2018). The effects of chronic stress on the human brain: from neurotoxicity to vulnerability. Annu. Rev. Neurosci. 41, 417–439. doi: 10.1146/annurev-neuro-080317-061727, 29421159

[ref56] Manzano G. (2002). Burnout y engagement: Relación con el desempeño, madurez profesional y tendencia al abandono de los estudiantes. Rev. Psicol. Soc. 17, 237–249. doi: 10.1174/02134740260372973

[ref57] Marcia J. E. (1980). Identity in adolescence. Handb. Adolesc. Psychol. 9, 159–187.

[ref58] Marcia J. E. Waterman A. S. Matteson D. R. Archer S. L. Orlofsky J. L. (2012). Ego Identity: A Handbook for Psychosocial Research. Heidelberg: Springer.

[ref59] Marsollier R. G. (2019). Análisis del modelo burnout-engagement en empleados públicos. Psicogente 22, 1–18. doi: 10.17081/psico.22.41.3311

[ref60] McEwen B. S. (2017). Neurobiological and systemic effects of chronic stress. Neuron 95, 267–279. doi: 10.1016/j.neuron.2017.07.005, 28856337 PMC5573220

[ref61] Munn Z. Peters M. D. J. Stern C. Tufanaru C. McArthur A. Aromataris E. (2018). Systematic review or scoping review? Guidance for authors when choosing between a systematic or scoping review approach. BMC Med. Res. Methodol. 18:143. doi: 10.1186/s12874-018-0611-x, 30453902 PMC6245623

[ref62] Neumann M. Edelhäuser F. Tauschel D. Fischer M. R. Wirtz M. Woopen C. . (2011). Empathy decline and its reasons: a systematic review of studies with medical students and residents. Acad. Med. 86, 996–1009. doi: 10.1097/ACM.0b013e318221e615, 21670661

[ref63] Núñez Lira L. A. Valentín Loayza J. E. Alfaro Mendives K. L. Bonilla Dulanto E. K. (2020). Governance, political representation and democratic disaffection in Peru [Gobernanza, representación política y desafección democrática en el Perú]. Rev. Venez. Gerenc. 25, 1330–1346. doi: 10.37960/rvg.v25i92.34265

[ref64] Orón Semper J. V. (2019). Neuropsicología de las emociones. Madrid: Piramide.

[ref65] Orón Semper J. V. (2023). Encuentro y crecimiento personal. Nueva propuesta educativa. Valencia: Ediciones Acompañando el Crecimiento.

[ref66] Otalora A. U. (2017). Populism as the vanguard of political disaffection in Europe: The phenomenon of “Podemos” political party in Spain [El populismo como vanguardia del desencanto político en Europa: El fenómeno «Podemos» en España]. Rev. Estud. Políticos 177, 213–255. doi: 10.18042/cepc/rep.177.07

[ref67] Pacheco C. (2021). Estrés en el trastorno límite de la personalidad: Una revisión de la vivencia particular desde una perspectiva microfenomenológica. Rev. Investig. Psicol. 24, 223–244. doi: 10.15381/rinvp.v28i1.20606

[ref68] Page M. J. McKenzie J. E. Bossuyt P. M. Boutron I. Hoffmann T. C. Mulrow C. D. . (2021). The PRISMA 2020 statement: an updated guideline for reporting systematic reviews. BMJ 372:n71. doi: 10.1136/bmj.n71, 33782057 PMC8005924

[ref69] Park C. L. Gutierrez I. A. (2013). Meaning violations and psychological distress following relationship loss: an application of the meaning-making model. J. Soc. Clin. Psychol. 32, 347–377. doi: 10.1521/jscp.2013.32.4.347

[ref70] Pascual Jimeno A. Conejero López S. (2015). La desmotivación del profesorado universitario y su relación con variables sociodemográficas, laborales y de personalidad. Apunt. Psicol. 33, 5–16. doi: 10.55414/vc3w5525

[ref72] Poteat V. P. Calzo J. P. Yoshikawa H. Kellogg D. Marx R. A. Richburg A. . (2025). Youth experiences in gender-sexuality alliances predict academic engagement but not disaffection through social-emotional wellbeing. Child Dev. 96, 847–864. doi: 10.1111/cdev.14209, 39658872 PMC11868679

[ref73] Prus R. Camara F. (2010). Love, friendship, and disaffection in Plato and Aristotle: toward a pragmatist analysis of interpersonal relationships. Qual. Sociol. Rev. 6, 29–62. doi: 10.18778/1733-8077.6.3.02

[ref74] Ramos A. G. (2024). Figures of disaffection in ethical life. Hegel and political subjectivity [Figuras de la desafección en la eticidad. Hegel y la subjetividad política]. Isegoría 70:1417. doi: 10.3989/isegoria.2024.70.1417

[ref75] Rivers N. (2021). The impact of corruption victimization on electoral alienation in Latin America. Public Integr. 24, 178–195. doi: 10.1080/10999922.2021.1885712

[ref76] Rosenberg M. J. Hovland C. I. (1960). “Cognitive, affective, and behavioral components of attitudes,” in Attitude Organization and Change: An Analysis of Consistency among Attitude Components, eds. Hovland C. I. Rosenberg M. J. (New Haven, CT: Yale University Press), 1–14.

[ref77] Rotenstein L. S. Ramos M. A. Torre M. Segal J. B. Peluso M. J. Guille C. . (2016). Prevalence of depression, depressive symptoms, and suicidal ideation among medical students: a systematic review and meta-analysis. JAMA 316, 2214–2236. doi: 10.1001/jama.2016.17324, 27923088 PMC5613659

[ref78] Saaty A. A. (2023). Correlations between expressing feelings, conveying thoughts, and gaining confidence when writing personal narratives in one's first and second language. World J. Engl. Lang. 13, 390–115. doi: 10.5430/wjel.v13n1p390

[ref79] Salvagioni D. A. J. Melanda F. N. Mesas A. E. González A. D. Gabani F. L. de Andra S. M. (2017). Physical, psychological and occupational consequences of job burnout: a systematic review. Front. Public Health 5:158. doi: 10.3389/fpubh.2017.00158, 28977041 PMC5627926

[ref80] Sánchez Llull D. Cerdà M. Ballester Brage L. (2015). Malestar social y malestar docente: Una investigación sobre el síndrome de desgaste profesional burnout y su incidencia socioeducativa. Rev. Aula 21, 245–250. doi: 10.14201/aula201521245257

[ref81] Selles J. F. (2015). ¿Es trascendental la antropología de Viktor E. Frankl? Madrid: Ápeiron.

[ref82] Serrano García J. Olmedo-Moreno E. M. Rakdani-Arif Billah F. Z. Expósito-López J. (2024). Tutorial action in primary and secondary education: a scientometric study for the period 2014 to 2024. Int. J. Learn. High. Educ. 32, 55–79. doi: 10.18848/2327-7955/CGP/v32i01/55-79

[ref83] Shaw R. J. Starr S. (2024). A systematic review on the overlap and distinction between anxiety and depression: insights from neurobiology and clinical practice. Front. Psych. 15:1510658. doi: 10.3389/fpsyt.2024.1510658/full

[ref84] Shonkoff J. P. Garner A. S. Siegel B. S. Dobbins M. I. Earls M. F. McGuinn L. . (2012). The lifelong effects of early childhood adversity and toxic stress. Pediatrics 129, e232–e246. doi: 10.1542/peds.2011-2663, 22201156

[ref85] Snape L. Atkinson C. (2015). Exploring and challenging pupil disaffection: an evaluation of a motivational interviewing-based intervention delivered by paraprofessionals. Pastoral Care Education 33, 69–82. doi: 10.1080/02643944.2015.1022207

[ref104] Upenieks L. (2024). Occupational Stressors and Flourishing among Roman Catholic Priests: The Eucharist as the “Source and Summit”. Review of Religious Research, 66. doi: 10.1177/0034673X241255058

[ref86] Vasan A. Mabey D. C. Chaudhri S. Brown Epstein H. A. Lawn S. D. (2017). Support and performance improvement for primary health care workers in low- and middle-income countries: a scoping review of intervention design and methods. Health Policy Plan. 32, czw144–czw452. doi: 10.1093/heapol/czw144, 27993961 PMC5400115

