# Supplementary material for: Systematic review and conceptualisation of disaffection for its accompaniment
Source: Front Psychol. 2026 Apr 23;17:1677767. doi: 10.3389/fpsyg.2026.1677767 (PMC13149428; doi:10.3389/fpsyg.2026.1677767)
Supplement: Supplementary file 1 [file Supplementary_file_1.DOCX]

***Supplementary Material***

**Index:**

Supplementary Material: Search Strategies. Pag 1.

Supplementary Material: Characteristics of the included articles. Pag. 2

Supplementary Material: JBI Critical Appraisal Tools Assesment. Pag 10

Supplementary Data: PRISMA Checklist. Pag 41.

1. **Supplementary Material: Search Strategies**

1.1. Web of Science

Document Types: Article.

Languages: English or Spanish.

Web of Science Index: Social Sciences Citation Index (SSCI) or Arts & Humanities Citation Index (A&HCI).

Web of Science Categories: Political Science or Education Educational Research or Psychology Educational or History or Communication or Sociology or Literature or Psychology Multidisciplinary or Economics or Psychology or Psychology Clinical or Family Studies or Social Sciences Interdisciplinary or Film Radio Television or Sport Sciences or Area Studies or Cultural Studies or International Relations or Music or Philosophy or Psychology Social or Anthropology or Education Scientific Disciplines or Environmental Sciences or Humanities Multidisciplinary or Language Linguistics or Literary Reviews or Literary Theory Criticism or Medieval Renaissance Studies or Psychology Applied or Psychology Developmental or Public Administration or Public Environmental Occupational Health or Social Work or Business or Development Studies or Environmental Studies or Ethnic Studies or Health Care Sciences Services or Law or Linguistics or Literature British Isles or Literature German Dutch Scandinavian or Literature Romance or Nursing or Psychology Mathematical or Religion or Social Issues or Theater or Industrial Relations Labor.

Publication Years: 2000 or 2001 or 2005 or 2003 or 2006 or 2007 or 2008 or 2009 or 2025 or 2024 or 2022 or 2021 or 2023 or 2020 or 2019 or 2018 or 2017 or 2016 or 2015 or 2014 or 2013 or 2012 or 2011 or 2010.

Type; Open Access

1.2. Pubmed

("Disaffection"[Title]) AND ((fha[Filter]) AND (humans[Filter]) AND (2000:2025/12/12[pdat]) AND (english[Filter] OR spanish[Filter]))

1.3. Scopus

TITLE ( DISAFFECTION ) AND PUBYEAR > 2000 AND PUBYEAR < 2026 AND ( LIMIT-TO ( DOCTYPE , "ar" ) ) AND ( LIMIT-TO ( LANGUAGE , "English" ) OR LIMIT-TO ( LANGUAGE , "Spanish" ) ) AND ( LIMIT-TO ( OA , "all" ) ) AND ( LIMIT-TO ( EXACTKEYWORD , "Disaffection" ) OR LIMIT-TO ( EXACTKEYWORD , "Political Disaffection" ) OR LIMIT-TO ( EXACTKEYWORD , "Democratic Disaffection" ) OR LIMIT-TO ( EXACTKEYWORD , "Satisfaction" ) OR LIMIT-TO ( EXACTKEYWORD , "Personal Satisfaction" ) OR LIMIT-TO ( EXACTKEYWORD , "Interpersonal Relations" ) OR LIMIT-TO ( EXACTKEYWORD , "Political Attitudes" ) OR LIMIT-TO ( EXACTKEYWORD , "Desafección Política" ) OR LIMIT-TO ( EXACTKEYWORD , "Cynicism" ) OR LIMIT-TO ( EXACTKEYWORD , "Apathy" ) OR LIMIT-TO ( EXACTKEYWORD , "Affection" ) ) AND ( LIMIT-TO ( SRCTYPE , "j" ) ) AND ( LIMIT-TO ( PUBSTAGE , "final" ) )

1. **Supplementary Material: Characteristics of the included articles**

| Study | Type of Study | Context | Country or Continent |
| --- | --- | --- | --- |
| Nuñez Lira et al., 2020 | Cross-Sectional Studies | Politics, society and citizenship | Peru |
| Allan, 2014 | Quasi-Experimental Study | Education | United Kingdom |
| Otalora, 2017 | Textual Evidence – Narrative | Politics, society and citizenship | Spain |
| Ramos, 2024 | Textual Evidence – Expert Opinion | Politics, society and citizenship | Spain |
| Arias, 2024 | Textual Evidence – Expert Opinion | Education | Spain |
| Prus & Camara, 2010 | Qualitative Research | Personal relationships | Greece |
| Henry & Thorsen, 2020 | Qualitative Research | Education | Sweden |
| Yamamoto & Kushin, 2014 | Analytical Cross-Sectional Study | Politics, society and citizenship | United States of America |
| Megías, 2020 | Analytical Cross-Sectional Study | Politics, society and citizenship | Spain |
| Curran et al., 2016 | Cohort Studies | Sport and physical activity | United Kingdom |
| Carrillo, 2017 | Analytical Cross-Sectional Study | Politics, society and citizenship | Central and South America |
| Allan & Duckworth, 2018 | Qualitative Research | Education | United Kingdom |
| Poteat et al., 2025 | Cohort Studies | Education | United States of America |
| Harber, 2008 | Textual Evidence – Expert Opinion | Education | United Kingdom |
| Fernandez et al., 2013 | Analytical Cross-Sectional Study | Education | Spain |
| Rodríguez-Medellín et al., 2020 | Diagnostic Test Accuracy Studies | Sport and physical activity | Mexico |
| González & Paoloni, 2014 | Analytical Cross-Sectional Study | Education | Spain |
| Cárdenas-Ruiz, 2022 | Analytical Cross-Sectional Study | Politics, society and citizenship | Colombia |
| Snape & Atkinson, 2015 | Quasi-Experimental Study | Religion | United Kingdom |
| Pešić et al., 2021 | Analytical Cross-Sectional Study | Politics, society and citizenship | Serbia |
| Villalobos, 2007 | Qualitative Research | Politics, society and citizenship | Chile |
| Fierro & Carbajal, 2022 | Analytical Cross-Sectional Study | Politics, society and citizenship | Peru |
| Lozano & Moya, 2016 | Qualitative Research | Politics, society and citizenship | Spain |
| Arango & Medina 2019 | Qualitative Research | Politics, society and citizenship | Colombia |
| Pizarro, 2018 | Qualitative Research | Politics, society and citizenship | Chile |
| Bright, 2011 | Qualitative Research | Education | United Kingdom |
| Snape & Atkinson, 2016 | Textual Evidence – Expert Opinion | Education | United Kingdom |
| Curranet al., 2015 | Systematic Reviews and Research Syntheses | Health and professions | Global |
| Megías & Moreno, 2016 | Textual Evidence – Narrative | Politics, society and citizenship | Europe |
| Boswell et al., 2016 | Qualitative Research | Politics, society and citizenship | United Kingdom |
| Pechenkina & Aeschliman, 2017 | Textual Evidence - Opinion | Education | Australia |
| O’Brien, 2003 | Qualitative Research | Education | United Kingdom |
| Manning & Holmes, 2013 | Textual Evidence - Opinion | Politics, society and citizenship | United States of America |
| Weiss & Livingston, 2020 | Analytical Cross-Sectional Study | Politics, society and citizenship | Europe |
| Paoloni & Rinaudo, 2017 | Analytical Cross-Sectional Study | Education | Argentina |
| Mainardes & gandin, 2013 | Qualitative Research | Education | Brazil |
| Solhaug, 2006 | Analytical Cross-Sectional Study | Education | Noruega |
| Herrera & Torres, 2006 | Text and Opinion | Education | Central and South America |
| Addison et al., 2023 | Analytical Cross-Sectional Study | Politics, society and citizenship | Germany |
| Megías et al., 2022 | Analytical Cross-Sectional Study | Politics, society and citizenship | Europe |
| Bakker et al., 2020 | Analytical Cross-Sectional Study | Politics, society and citizenship | Europe |
| Garrett et al., 2008 | Analytical Cross-Sectional Study | Health and professions | United States of America |
| Järvinen et al., 2025 | Analytical Cross-Sectional Study | Education | Finland |
| Fontaneda et al., 2018 | Analytical Cross-Sectional Study | Politics, society and citizenship | Spain |
| Boaler et al., 2000 | Qualitative Research | Education | United Kingdom |
| Schulte-Cloos et al., 2022 | Analytical Cross-Sectional Study | Politics, society and citizenship | Germany |
| Sarr et al., 2020 | Qualitative Research | Politics, society and citizenship | Senegal |
| Sandford et al. 2008 | Text and Opinion | Sport and physical activity | United Kingdom |
| Rozas-Bugueño, 2024 | Text and Opinion | Politics, society and citizenship | Chile |
| Nardi et al., 2003 | Qualitative Research | Education | Italy |
| Skinner, 2022 | Text and Opinion | Politics, society and citizenship | United Kingdom |
| Boswell et al., 2019 | Text and Opinion | Politics, society and citizenship | United Kingdom |
| Sakellariou et al., 2020 | Qualitative Research | Education | Greece |
| Ritosa, 2022 | Analytical Cross-Sectional Study | Education | Sweden |
| Curran et al., 2013 | Analytical Cross-Sectional Study | Sport and physical activity | Australia |
| Flanagan, 2010 | Text and Opinion | Education | United States of America |
| Stewart et al., 2007 | Analytical Cross-Sectional Study | Politics, society and citizenship | United States of America |
| Zaff et al., 2010 | Analytical Cross-Sectional Study | Politics, society and citizenship | United States of America |
| Ekman et al., 2012 | Analytical Cross-Sectional Study | Politics, society and citizenship | Sweden |
| Ganotice et al., 2022 | Analytical Cross-Sectional Study | Health and professions | Hong Kong |
| Rotger, 2015 | Text and Opinion | Health and professions | Spain |
| Burić et al., 2024 | Analytical Cross-Sectional Study | Education | Croatia |
| Galand & Hospel, 2013 | Analytical Cross-Sectional Study | Health and professions | Belgium |
| González et al., 2015 | Analytical Cross-Sectional Study | Education | Spain |
| Jones et al., 2006 | Text and Opinion | Politics, society and citizenship | Australia |
| Furrer, 2010 | Analytical Cross-Sectional Study | Personal relationships | United States of America |
| Knight et al., 2002 | Analytical Cross-Sectional Study | Health and professions | United States of America |

**References:**

Addison, John T.; Teixeira, Paulino; Grunau, Philipp; Bellmann, Lutz. “Works council 'disaffection' and establishment survivability.” Scottish Journal of Political Economy, vol. 70, no. 1, Feb. 2023, pp. 38–67. doi:10.1111/sjpe.12330.

Allan, D. (2014). Dealing with disaffection: The influence of work-based learning on 14–16-year-old students' attitudes to school. Empirical Research in Vocational Education and Training, 6(1), Article 6. https://doi.org/10.1186/s40461-014-0010-4

Allan, D., & Duckworth, V. (2018). Voices of disaffection: disengaged and disruptive youths or agents of change and self-empowerment? British Journal of Special Education, 45(1), 43–60. https://doi.org/10.1111/1467-8578.12201

Arango, G., & Medina, L. (2019). From disaffection to rebellion: Youth and political subjectivity in Colombia [De la desafección a la rebeldía: Juventud y subjetividad política en Colombia]. Estudios Políticos, 54, 103–129. https://doi.org/10.17533/udea.espo.n54a06

Arias, G. V. (2024). Arrogance, distrust and disaffection. An approach based on the epistemology of virtue [Arrogancia, desconfianza y desafección. Una aproximación desde la epistemología de la virtud]. Isegoría, (70), Article 1348. https://doi.org/10.3989/isegoria.2024.70.1348

Bakker, Ryan; Jolly, Seth; Polk, Jonathan. “Multidimensional incongruence, political disaffection, and support for anti-establishment parties.” Journal of European Public Policy, vol. 27, no. 2, 1 Feb. 2020, pp. 292–309. doi:10.1080/13501763.2019.1701534.

Boaler, Jo; Wiliam, Dylan; Brown, Margaret. “Students' Experiences of Ability Grouping — Disaffection, Polarisation and the Construction of Failure.” British Educational Research Journal, vol. 26, no. 5, Dec. 2000, pp. 631–648. doi:10.1080/713651583.

Boswell, J., Corbett, J., Rhodes, R. A. W., & Weller, P. (2016). Rethinking disaffection in ethnographic political studies: ‘Being’ cynical in the UK. Politics, 36(2), 147–162. https://doi.org/10.1177/0263395715618401

Boswell, John; Corbett, Jack; Dommett, Kate; Jennings, Will; Flinders, Matthew; Rhodes, R. A. W.; Wood, Matthew. “State of the field: What can political ethnography tell us about anti-politics and democratic disaffection?” European Journal of Political Research, vol. 58, no. 1, Feb. 2019, pp. 56–71. doi:10.1111/1475-6765.12270.

Bright, G. (2011). ‘Non-servile virtuosi’ in insubordinate spaces: School disaffection, refusal and resistance in a UK context. European Journal of Cultural Studies, 14(5), 567–582. https://doi.org/10.1177/1367549411419978

Burić, I., Huić, A., & Sorić, I. (2024). Are student engagement and disaffection important for teacher well-being? A longitudinal examination of between- and within-person effects. Journal of School Psychology, 103, 101289. https://doi.org/10.1016/j.jsp.2024.101289

Cárdenas Ruiz, J. D. (2022). Paradoxes and transformations of political participation in Bogota: Political disaffection and participation in the 2019 elections [Las paradojas y transformaciones de la participación política en Bogotá: desafección política y participación en las elecciones de 2019]. Comunicación y Sociedad (México), (19), Article e8324. https://doi.org/10.32870/cys.v2022.8324

Carrillo, A. M. (2017). The representative disaffection in Latin America [La desafección representativa en América Latina]. Andamios, 14(35), 17–41. https://doi.org/10.29092/uacm.v14i35.570

Curran, T., Hill, A. P., Appleton, P. R., Vallerand, R. J., & Standage, M. (2015). The psychology of passion: A meta-analytical review of a decade of research on intrapersonal outcomes. Motivation and Emotion, 39(5), 631–655. https://doi.org/10.1007/s11031-015-9503-0

Curran, T., Hill, A. P., Ntoumanis, N., Hall, H. K., & Jowett, G. E. (2016). A three-wave longitudinal test of self-determination theory's mediation model of engagement and disaffection in youth sport. Journal of Sport and Exercise Psychology, 38(1), 15–29. https://doi.org/10.1123/jsep.2015-0016

Curran, Thomas; Hill, Andrew P.; Niemiec, Christopher P. “A Conditional Process Model of Children's Behavioral Engagement and Behavioral Disaffection in Sport Based on Self-Determination Theory.” Journal of Sport & Exercise Psychology, vol. 35, no. 1, Feb. 2013, pp. 30–43. doi:10.1123/jsep.35.1.30.

Ekman, Joakim; Amnå, Erik. “Political participation and civic engagement: Towards a new typology.” Human Affairs, vol. 22, no. 3, 2012, pp. 283–300. doi:10.2478/s13374-012-0036-7.

Fernández, A. G., Paoloni, P. V., Rinaudo, M. C., & Donolo, D. (2013). Situational interest in Spanish language class on secondary education: Structural relations with engagement, disaffection, and performance [Interés situacional en clase de Lengua Española en secundaria: Relaciones estructurales con el compromiso, el desapego y el rendimiento]. Universitas Psychologica, 12(3), 753–766. https://doi.org/10.11144/Javeriana.UPSY12-3.iscl

Fierro, C., & Carbajal, J. (2022). Political disaffection among Peruvian youth: Between distrust and democratic commitment [Desafección política en jóvenes peruanos: Entre la desconfianza y el compromiso democrático]. Debates en Sociología, (57), 75–104. https://doi.org/10.18800/debatesensociologia.202202.003

Flanagan, Constance A.; Levine, Peter. “Civic engagement and the transition to adulthood.” The Future of Children, vol. 20, no. 1, Spring 2010, pp. 159–179. doi:10.1353/foc.0.0043.

Fontaneda, Javier Lorente; Sánchez-Vítores, Irene. “Disaffection at the Ballot Box: The 2015 General Election in Spain.” Revista Española de Investigaciones Sociológicas, no. 161, Jan.–Mar. 2018, pp. 41–62. doi:10.5477/cis/reis.161.41.

Full citation

Furrer, C. J. (2010). Capturing the friendship context with a collective property: Friendship group engagement vs. disaffection. Journal of Adolescence, 33(6), 853–867. https://doi.org/10.1016/j.adolescence.2010.07.003

Galand, B., & Hospel, V. (2013). Peer victimization and school disaffection: Exploring the moderation effect of social support and the mediation effect of depression. British Journal of Educational Psychology, 83(4), 569–590. https://doi.org/10.1111/j.2044-8279.2012.02077.xGaland, B., & Hospel, V. (2013). Peer victimization and school disaffection: Exploring the moderation effect of social support and the mediation effect of depression. British Journal of Educational Psychology, 83(4), 569–590. https://doi.org/10.1111/j.2044-8279.2012.02077.x

Ganotice, F. A., Chan, C. S., Chan, E. W. Y., Chan, S. K. W., Chan, L., Chan, S. C. S., Lam, A. H. Y., Leung, C. Y. F., Leung, S. C., Lin, X., Luk, P., Ng, Z. L. H., Shen, X., Tam, E. Y. T., Wang, R., Wong, G. H. Y., & Tipoe, G. L. (2022). Autonomous motivation predicts students' engagement and disaffection in interprofessional education: Scale adaptation and application. Nurse Education Today, 119, 105549. https://doi.org/10.1016/j.nedt.2022.105549

Garrett, R. Kelly; Danziger, James N. “Disaffection or expected outcomes: Understanding personal Internet use during work.” Journal of Computer-Mediated Communication, vol. 13, no. 4, Jul. 2008, pp. 937–958. doi:10.1111/j.1083-6101.2008.00425.x.

González, A., & Paoloni, P. V. (2014). Self-determination, behavioral engagement, disaffection, and academic performance: A mediational analysis. Spanish Journal of Psychology, 17(2), Article e82. https://doi.org/10.1017/sjp.2014.82

González, A., Faílde Garrido, J. M., Rodríguez Castro, Y., & Carrera Rodríguez, M. V. (2015). Class anxiety in secondary education: Exploring structural relations with perceived control, engagement, disaffection, and performance. The Spanish Journal of Psychology, 18, E68. https://doi.org/10.1017/sjp.2015.70

Harber, C. (2008). Perpetrating disaffection: Schooling as an international problem. Educational Studies, 34(5), 457–467. https://doi.org/10.1080/03055690802288445

Henry, A., & Thorsen, C. (2020). Disaffection and agentic engagement: ‘Redesigning’ activities to enable authentic self-expression. Language Teaching Research, 24(4), 456–475. https://doi.org/10.1177/1362168818795976

Herrera, L., & Torres, C. A. (2006). Cultures of politics/politics of cultures: Re-visioning Latin American education. International Studies in Sociology of Education, 16(2), 131–144. https://doi.org/10.1080/09620210600818803

Järvinen, Jussi; Hietajärvi, Lauri; Ketonen, Elina E.; Salmela-Aro, Katariina. “Instruction and task appraisals as antecedents of momentary engagement and disaffection.” Educational Psychology, early access, 14 May 2025. doi:10.1080/01443410.2025.2501144.

Jones, J. A., Meehan-Andrews, T. A., Smith, K. B., Humphreys, J. S., Griffin, L., & Wilson, B. (2006). “There's no point in complaining, nothing changes”: Rural disaffection with complaints as an improvement method. Australian Health Review, 30(3), 322–332. https://doi.org/10.1071/ah060322

Knight, T. T., Richardson, J. D., & Kalbfleisch, J. H. (2002). Career disaffection among surgeons in the era of managed care. The American Surgeon, 68(6), 519–523. doi: 10.1177/000313480206800603

Lozano, J. F., & Moya, F. (2016). Disaffection and political cynicism in Spanish university students: Between indignation and disengagement [Desafección y cinismo político en universitarios españoles: Entre la indignación y el desapego]. Revista Española de Ciencia Política, (40), 45–66. https://doi.org/10.21308/recp.40.03

Mainardes, J., & Gandin, L. A. (2013). The dialectics of engagement and disaffection in educational reform: The case of the Citizen School Project in Brazil. Policy Futures in Education, 11(5), 546–560. https://doi.org/10.2304/pfie.2013.11.5.546

Manning, N., & Holmes, M. (2013). Political engagement and the ‘youth problem’: A critical overview. Sociology Compass, 7(1), 14–26. https://doi.org/10.1111/soc4.12010

Megías, A. (2020). Changes in the nature of a decade-long crisis of disaffection [Una década de crisis desafecta: Los cambios en su naturaleza]. Revista Española de Investigaciones Sociológicas, 169, 103–122. https://doi.org/10.5477/cis/reis.169.103

Megías, Adrián; Moreno, Cristina. “Political Disaffection in European Countries near Spain: A Stable Attitude?” Revista Española de Investigaciones Sociológicas, no. 179, Jul.–Sep. 2022, pp. 103–122. doi:10.5477/cis/reis.179.103.

Megías, I., & Moreno, A. (2016). Young people and political engagement in Europe: Disaffection or adaptation? Youth & Policy, (116), 1–18. https://www.youthandpolicy.org/articles/young-people-political-engagement-europe/

Nardi, Elena; Steward, Sue. “Is mathematics TIRED? A profile of quiet disaffection in the secondary mathematics classroom.” British Educational Research Journal, vol. 29, no. 3, Jun. 2003, pp. 345–367. doi:10.1080/01411920301852.

Núñez Lira, L. A., Valentín Loayza, J. E., Alfaro Mendives, K. L., & Bonilla Dulanto, E. K. (2020). Governance, political representation and democratic disaffection in Peru [Gobernanza, representación política y desafección democrática en el Perú]. Revista Venezolana de Gerencia, 25(92), 1330–1346. https://doi.org/10.37960/rvg.v25i92.34265

O’Brien, M. (2003). “That girl’s wearing army stuff!”: Girls, gender and political disaffection. Youth & Society, 35(2), 231–254. https://doi.org/10.1177/0044118X03255060

Otalora, A. U. (2017). Populism as the vanguard of political disaffection in Europe: The phenomenon of “Podemos” political party in Spain [El populismo como vanguardia del desencanto político en Europa: El fenómeno «Podemos» en España]. Revista de Estudios Políticos, (177), 213–255. https://doi.org/10.18042/cepc/rep.177.07

Paoloni, P. V., & Rinaudo, M. C. (2017). Academic disaffection and achievement emotions in university students. Journal of Educational Psychology-Propositos y Representaciones, 5(1), 211–242. https://doi.org/10.20511/pyr2017.v5n1.138

Pechenkina, E., & Aeschliman, C. (2017). Creating an engaged learning experience for students through peer-assisted learning in STEM disciplines: A practice report. International Journal of Educational Research, 86, 141–148. https://doi.org/10.1016/j.ijer.2017.10.002

Pešić, J., Birešev, A., & Petrović Trifunović, T. (2021). Political disaffection and disengagement in Serbia [Otklon od politike i dezangažman u Srbiji]. Sociologija, 63(2), 355–380. https://doi.org/10.2298/SOC2102355P

Pizarro, R. (2018). Disaffection and political subjectivities: Approaches from youth experiences in Santiago [Desafección y subjetividades políticas: Aproximaciones desde experiencias juveniles en Santiago]. Última Década, 26(49), 11–36. https://doi.org/10.4067/S0718-22362018000100011

Poteat, V. P., Calzo, J. P., Yoshikawa, H., Kellogg, D., Marx, R. A., Richburg, A., & Lipkin, A. (2025). Youth experiences in gender–sexuality alliances predict academic engagement but not disaffection through social–emotional wellbeing. Child Development, 96(2), 847–864. https://doi.org/10.1111/cdev.14209

Prus, R., & Camara, F. (2010). Love, friendship, and disaffection in Plato and Aristotle: Toward a pragmatist analysis of interpersonal relationships. Qualitative Sociology Review, 6(3), 29–62. https://doi.org/10.18778/1733-8077.6.3.02

Ramos, A. G. (2024). Figures of disaffection in ethical life. Hegel and political subjectivity [Figuras de la desafección en la eticidad. Hegel y la subjetividad política]. Isegoría, (70), Article 1417. https://doi.org/10.3989/isegoria.2024.70.1417

Ritosa, Andrea. “Validation of the School Engagement Questionnaire Engagement Versus Disaffection With Learning: Teacher Report in Swedish 6th Graders.” Journal of Psychoeducational Assessment, vol. 40, no. 4, Jul. 2022, pp. 549–558. doi:10.1177/07342829211067750.

Rodríguez-Medellín, R., Zamarripa, J., Marentes-Castillo, M., Otero-Saborido, F., Baños, R., & Morquecho-Sánchez, R. (2020). Mexican validation of the engagement and disaffection in physical education scale. International Journal of Environmental Research and Public Health, 17(6), Article 1821. https://doi.org/10.3390/ijerph17061821

Rotger, N. (2025). Narrating loneliness: Isolation, disaffection, and the contemporary novel. Journal of Medical Humanities, 46(2), 221–234. https://doi.org/10.1007/s10912-024-09855-z

Rozas-Bugueño, Joaquín. “Between Hope and Disaffection: The Chilean Constitution-Making Process and the Intermediation Crisis.” PS: Political Science & Politics, vol. 57, no. 2, Apr. 2024, pp. 274–281. doi:10.1017/S1049096523001130.

Sakellariou, Maria; Tsiara, Efthymia. “Student Disaffection: The Contribution of Greek In-service Kindergarten Teachers in Engaging Each Preschooler in Learning.” Behavioral Sciences, vol. 10, no. 2, Feb. 2020, article 51. doi:10.3390/bs10020051.

Sandford, Rachel A.; Duncombe, Rebecca; Armour, Kathy M. “The role of physical activity/sport in tackling youth disaffection and anti-social behaviour.” Educational Review, vol. 60, no. 4, 2008, pp. 419–435. doi:10.1080/00131910802393464.

Sarr, Birame; González-Hernández, Matías Manuel; Boza-Chirino, José; de León, Javier. “Understanding Communities' Disaffection to Participate in Tourism in Protected Areas: A Social Representational Approach.”

Schulte-Cloos, Julia; Leininger, Arndt. “Electoral participation, political disaffection, and the rise of the populist radical right.” Party Politics, vol. 28, no. 3, May 2022, pp. 431–443. doi:10.1177/1354068820985186.

Skinner, Stephen. “Inciting Military Disaffection in Interwar Britain and Fascist Italy: Security, Crime and Authoritarian Law.” Oxford Journal of Legal Studies, vol. 42, no. 2, May 2022, pp. 578–605. doi:10.1093/ojls/gqab036.

Snape, L., & Atkinson, C. (2015). Exploring and challenging pupil disaffection: an evaluation of a motivational interviewing-based intervention delivered by paraprofessionals. Pastoral Care in Education, 33(2), 69–82. https://doi.org/10.1080/02643944.2015.1022207

Snape, L., & Atkinson, C. (2016). The evidence base for pupil disaffection: An evidence-informed conceptual framework for practitioners. Educational and Child Psychology, 33(2), 32–49. https://www.researchgate.net/publication/308783223

Solhaug, T. (2006). Knowledge and self-efficacy as predictors of political participation and disaffection among Norwegian adolescents. Policy Futures in Education, 4(3), 259–270. https://doi.org/10.2304/pfie.2006.4.3.259

Stewart, Abigail J.; Settles, Isis H.; Winter, David G. “Women's leadership in the United States: An examination of perceptions and the complexity of social identity.” American Psychologist, vol. 62, no. 4, May–Jun. 2007, pp. 415–428. doi:10.1037/0003-066X.62.4.415.

Villalobos, C. (2007). Disaffection and political participation in Chilean youth [Desafección y participación política en jóvenes chilenos]. Revista Latinoamericana de Ciencias Sociales, Niñez y Juventud, 5(2), 1–26. https://doi.org/10.11600/1692715x.52206

Weiss, A., & Livingston, M. (2020). Disaffection or adaptation? Youth civic attitudes in times of democratic crisis. Journal of Youth Studies, 23(4), 446–462. https://doi.org/10.1080/13676261.2019.1620921

Yamamoto, M., & Kushin, M. J. (2014). More harm than good? Online media use and political disaffection among college students in the 2008 election. Journal of Computer-Mediated Communication, 19(3), 430–445. https://doi.org/10.1111/jcc4.12046

Zaff, Jonathan F.; Boyd, Michelle J.; Li, Yibing; Lerner, Jacqueline V.; Lerner, Richard M. “Active and engaged citizenship: Multi-group and longitudinal factorial analysis of an integrated construct of civic engagement.” Journal of Youth and Adolescence, vol. 39, no. 7, Jul. 2010, pp. 736–750. doi:10.1007/s10964-010-9541-6.

1. **Supplementary Material: JBI Critical Appraisal Tools Assesment**

Article 1: Núñez Lira, L. A., Valentín Loayza, J. E., Alfaro Mendives, K. L., & Bonilla Dulanto, E. K. (2020). Governance, political representation and democratic disaffection in Peru. Revista Venezolana de Gerencia, 25(92), 1330–1346. https://doi.org/10.37960/rvg.v25i92.34265

Checklist Application (JBI Analytical Cross-Sectional Studies):

- Inclusion criteria clearly defined: Yes
- Study subjects and setting described in detail: Yes
- Exposure measured validly and reliably: Yes
- Objective, standard criteria used for measurement: Yes
- Confounding factors identified: Unclear
- Strategies to deal with confounding factors stated: No
- Outcomes measured validly and reliably: Yes
- Appropriate statistical analysis used: Yes

Conclusion: Included. The article meets most methodological criteria, clearly defining sample inclusion and providing rigorous and valid measurements, despite limitations concerning explicit strategies to manage confounding factors.

Article 2: Allan, D. (2014). Dealing with disaffection: The influence of work-based learning on 14–16-year-old students' attitudes to school. Empirical Research in Vocational Education and Training, 6(1), Article 6. https://doi.org/10.1186/s40461-014-0010-4

Article Type: Quasi-Experimental Study

Checklist Application (JBI Quasi-Experimental Studies):

- Clear causal relationship (temporal precedence): Yes
- Presence of control group: Yes
- Similar participants across comparisons: Yes
- Similar treatment apart from intervention: Yes
- Multiple pre- and post-intervention measurements: Yes
- Outcome measurement consistency: Yes
- Reliable outcome measurement methods: Yes
- Adequate follow-up and analysis of differences: Yes
- Appropriate statistical analysis: Yes

Conclusion: Included. Strong methodological rigor, presence of control groups, repeated measures, and thorough statistical analysis justify inclusion.

Article 3: Otalora, A. U. (2017). Populism as the vanguard of political disaffection in Europe: The phenomenon of "Podemos" political party in Spain. Revista de Estudios Políticos, (177), 213–255. https://doi.org/10.18042/cepc/rep.177.07

Checklist Application (JBI Textual Evidence – Narrative):

- Credible/appropriate source: Yes
- Context clearly explained: Yes
- Logical event sequence: Yes
- Similar conclusions by reviewer and narrator: Yes
- Conclusions flow logically from narrative: Yes
- Narrative clearly identifiable: Yes

Conclusion: Included. Clear coherence and rigorous contextualization of narrative elements align logically, meeting all JBI criteria.

Article 4: Ramos, A. G. (2024). Figures of disaffection in ethical life. Hegel and political subjectivity. Isegoría, (70), Article 1417. https://doi.org/10.3989/isegoria.2024.70.1417

Checklist Application (JBI Textual Evidence – Expert Opinion):

- Source of opinion clearly identified: Yes
- Source has standing in expertise field: Yes
- Interests of relevant population central: Yes
- Logical argumentation clearly defended: Yes
- Reference to extant literature: Yes
- Incongruence with literature defended logically: Not applicable

Conclusion: Included. Well-established expert credentials, coherent logical arguments, and solid references to existing literature make it a robust expert opinion piece.

Article 5: Arias, G. V. (2024). Arrogance, distrust and disaffection. An approach based on the epistemology of virtue. Isegoría, (70), Article 1348. https://doi.org/10.3989/isegoria.2024.70.1348

Checklist Application (JBI Textual Evidence – Expert Opinion):

- Source of opinion clearly identified: Yes
- Source has standing in expertise field: Yes
- Interests of relevant population central: Yes
- Logical argumentation clearly defended: Yes
- Reference to extant literature: Yes
- Incongruence with literature defended logically: Not applicable

Conclusion: Included. The article demonstrates scholarly rigor, clearly identified authorship, sound logical arguments, and robust references to relevant epistemological literature.

Article 6: Prus, R., & Camara, F. (2010). Love, friendship, and disaffection in Plato and Aristotle: Toward a pragmatist analysis of interpersonal relationships. Qualitative Sociology Review, 6(3), 29–62. https://doi.org/10.18778/1733-8077.6.3.02

Checklist Application (JBI Qualitative Research):

- Congruity between philosophical perspective and methodology: Yes
- Congruity between methodology and research question/objectives: Yes
- Congruity between methodology and data collection methods: Yes
- Congruity between methodology and data analysis representation: Yes
- Congruity between methodology and interpretation of results: Yes
- Researcher location culturally/theoretically identified: Yes
- Influence of researcher on research addressed: Yes
- Participants adequately represented: Not applicable (theoretical paper)
- Ethical approval or ethical considerations addressed: Not applicable
- Conclusions drawn flow from analysis/interpretation: Yes

Conclusion: Included. Article demonstrates comprehensive congruity between philosophical framing, methodology, analysis, and interpretation, fully meeting qualitative research criteria.

Article 7: Henry, A., & Thorsen, C. (2020). Disaffection and agentic engagement: ‘Redesigning’ activities to enable authentic self-expression. Language Teaching Research, 24(4), 456–475. https://doi.org/10.1177/1362168818795976

Checklist Application (JBI Qualitative Research):

- Congruity between philosophical perspective and methodology: Yes
- Congruity between methodology and research question/objectives: Yes
- Congruity between methodology and data collection methods: Yes
- Congruity between methodology and data analysis representation: Yes
- Congruity between methodology and interpretation of results: Yes
- Researcher location culturally/theoretically identified: Yes
- Influence of researcher on research addressed: Yes
- Participants adequately represented: Yes
- Ethical approval or ethical considerations addressed: Yes
- Conclusions drawn flow from analysis/interpretation: Yes

Conclusion: Included. Meticulous alignment of research methods, theoretical grounding, and ethical considerations confirms high-quality qualitative research standards.

Article 8: Yamamoto, M., & Kushin, M. J. (2014). More harm than good? Online media use and political disaffection among college students in the 2008 election. Journal of Computer-Mediated Communication, 19(3), 430–445. https://doi.org/10.1111/jcc4.12046

Checklist Application (JBI Analytical Cross-Sectional Studies):

- Inclusion criteria clearly defined: Yes
- Study subjects and setting described in detail: Yes
- Exposure measured validly and reliably: Yes
- Objective, standard criteria used for measurement: Yes
- Confounding factors identified: Yes
- Strategies to deal with confounding factors stated: Yes
- Outcomes measured validly and reliably: Yes
- Appropriate statistical analysis used: Yes

Conclusion: Included. Comprehensive methodological rigor and clearly articulated statistical procedures satisfy all relevant checklist criteria.

Article 9: Megías, A. (2020). Changes in the nature of a decade-long crisis of disaffection. Revista Española de Investigaciones Sociológicas, 169, 103–122. https://doi.org/10.5477/cis/reis.169.103

Checklist Application (JBI Analytical Cross-Sectional Studies):

- Inclusion criteria clearly defined: Yes
- Study subjects and setting described in detail: Yes
- Exposure measured validly and reliably: Yes
- Objective, standard criteria used for measurement: Yes
- Confounding factors identified: Yes
- Strategies to deal with confounding factors stated: Yes
- Outcomes measured validly and reliably: Yes
- Appropriate statistical analysis used: Yes

Conclusion: Included. Meets rigorous methodological criteria, clearly defining its sample and employing thorough statistical analyses.

Article 10: Curran, T., Hill, A. P., Ntoumanis, N., Hall, H. K., & Jowett, G. E. (2016). A three-wave longitudinal test of self-determination theory's mediation model of engagement and disaffection in youth sport. Journal of Sport and Exercise Psychology, 38(1), 15–29. https://doi.org/10.1123/jsep.2015-0016

Checklist Application (JBI Cohort Studies):

- Groups similar, recruited from same population: Yes
- Exposures measured similarly: Yes
- Exposure measurement valid/reliable: Yes
- Confounding factors identified: Yes
- Strategies to deal with confounding stated: Yes
- Participants free of outcome at study start: Yes
- Outcomes measured validly/reliably: Yes
- Follow-up time sufficient: Yes
- Complete follow-up or justified losses: Yes
- Strategies for incomplete follow-up: Yes
- Appropriate statistical analysis: Yes

Conclusion: Included. Robust study design, clearly articulated methodological steps, effective management of confounding factors, and comprehensive statistical analysis.

Article 11: Carrillo, A. M. (2017). The representative disaffection in Latin America. Andamios, 14(35), 17–41. https://doi.org/10.29092/uacm.v14i35.570

Checklist Application (JBI Analytical Cross-Sectional Studies):

- Inclusion criteria clearly defined: Yes
- Study subjects and setting described in detail: Yes
- Exposure measured validly and reliably: Yes
- Objective, standard criteria used for measurement: Yes
- Confounding factors identified: Yes
- Strategies to deal with confounding factors stated: No
- Outcomes measured validly and reliably: Yes
- Appropriate statistical analysis used: Yes

Conclusion: Included. Article clearly defines criteria, setting, and reliable measurements, despite lacking explicit strategies to address confounding factors.

Article 12: Allan, D., & Duckworth, V. (2018). Voices of disaffection: disengaged and disruptive youths or agents of change and self-empowerment? British Journal of Special Education, 45(1), 43–60. https://doi.org/10.1111/1467-8578.12201

Checklist Application (JBI Qualitative Research):

- Congruity between philosophical perspective and methodology: Yes
- Congruity between methodology and research question/objectives: Yes
- Congruity between methodology and data collection methods: Yes
- Congruity between methodology and data analysis representation: Yes
- Congruity between methodology and interpretation of results: Yes
- Researcher location culturally/theoretically identified: Yes
- Influence of researcher on research addressed: Yes
- Participants adequately represented: Yes
- Ethical approval or ethical considerations addressed: Yes
- Conclusions drawn flow from analysis/interpretation: Yes

Conclusion: Included. Comprehensive qualitative research with clear theoretical framing, robust methodological congruity, and ethical considerations.

Article 13: Poteat, V. P., Calzo, J. P., Yoshikawa, H., Kellogg, D., Marx, R. A., Richburg, A., & Lipkin, A. (2025). Youth experiences in gender–sexuality alliances predict academic engagement but not disaffection through social–emotional wellbeing. Child Development, 96(2), 847–864. https://doi.org/10.1111/cdev.14209

Checklist Application (JBI Cohort Studies):

- Groups similar, recruited from same population: Yes
- Exposures measured similarly: Yes
- Exposure measurement valid/reliable: Yes
- Confounding factors identified: Yes
- Strategies to deal with confounding stated: Yes
- Participants free of outcome at study start: Yes
- Outcomes measured validly/reliably: Yes
- Follow-up time sufficient: Yes
- Complete follow-up or justified losses: Yes
- Strategies for incomplete follow-up: Yes
- Appropriate statistical analysis: Yes

Conclusion: Included. Rigorous design, clear cohort management, and effective control of confounding variables with comprehensive statistical support.

Article 14: Harber, C. (2008). Perpetrating disaffection: Schooling as an international problem. Educational Studies, 34(5), 457–467. https://doi.org/10.1080/03055690802288445

Checklist Application (JBI Textual Evidence – Expert Opinion):

- Source of opinion clearly identified: Yes
- Source has standing in expertise field: Yes
- Interests of relevant population central: Yes
- Logical argumentation clearly defended: Yes
- Reference to extant literature: Yes
- Incongruence with literature defended logically: Not applicable

Conclusion: Included. Expert opinion clearly articulated, logically consistent, and well-grounded in existing literature.

Article 15: Fernández, A. G., Paoloni, P. V., Rinaudo, M. C., & Donolo, D. (2013). Situational interest in Spanish language class on secondary education: Structural relations with engagement, disaffection, and performance. Universitas Psychologica, 12(3), 753–766. https://doi.org/10.11144/Javeriana.UPSY12-3.iscl

Checklist Application (JBI Analytical Cross-Sectional Studies):

- Inclusion criteria clearly defined: Yes
- Study subjects and setting described in detail: Yes
- Exposure measured validly and reliably: Yes
- Objective, standard criteria used for measurement: Yes
- Confounding factors identified: Yes
- Strategies to deal with confounding factors stated: Yes
- Outcomes measured validly and reliably: Yes
- Appropriate statistical analysis used: Yes

Conclusion: Included. Meets all methodological criteria, clearly defining its analytical framework, reliably measuring outcomes, and employing rigorous statistical methods.

Article 16: Rodríguez-Medellín, R., Zamarripa, J., Marentes-Castillo, M., Otero-Saborido, F., Baños, R., & Morquecho-Sánchez, R. (2020). Mexican validation of the engagement and disaffection in physical education scale. International Journal of Environmental Research and Public Health, 17(6), Article 1821. https://doi.org/10.3390/ijerph17061821

Checklist Application (JBI Diagnostic Test Accuracy Studies):

- Consecutive or random sample enrollment: Yes
- Case-control design avoided: Yes
- Inappropriate exclusions avoided: Yes
- Index test results interpreted without knowledge of the reference standard: Not applicable
- Threshold pre-specified: Not applicable
- Reference standard likely correct classification: Yes
- Reference standard results interpreted without knowledge of index test: Not applicable
- Appropriate interval between tests: Not applicable
- Same reference standard applied to all patients: Yes
- All patients included in analysis: Yes

Conclusion: Included. Strong methodological validation with proper sampling and comprehensive analytical approach, meeting key criteria.

Article 17: González, A., & Paoloni, P. V. (2014). Self-determination, behavioral engagement, disaffection, and academic performance: A mediational analysis. Spanish Journal of Psychology, 17(2), Article e82. https://doi.org/10.1017/sjp.2014.82

Checklist Application (JBI Analytical Cross-Sectional Studies):

- Inclusion criteria clearly defined: Yes
- Study subjects and setting described in detail: Yes
- Exposure measured validly and reliably: Yes
- Objective, standard criteria used for measurement: Yes
- Confounding factors identified: Yes
- Strategies to deal with confounding factors stated: Yes
- Outcomes measured validly and reliably: Yes
- Appropriate statistical analysis used: Yes

Conclusion: Included. Comprehensive methodology with rigorous measurements and effective confounding controls ensure strong validity.

Article 18: Cárdenas Ruiz, J. D. (2022). Paradoxes and transformations of political participation in Bogota: Political disaffection and participation in the 2019 elections. Comunicación y Sociedad (México), (19), Article e8324. https://doi.org/10.32870/cys.v2022.8324

Checklist Application (JBI Analytical Cross-Sectional Studies):

- Inclusion criteria clearly defined: Yes
- Study subjects and setting described in detail: Yes
- Exposure measured validly and reliably: Yes
- Objective, standard criteria used for measurement: Yes
- Confounding factors identified: Yes
- Strategies to deal with confounding factors stated: Yes
- Outcomes measured validly and reliably: Yes
- Appropriate statistical analysis used: Yes

Conclusion: Included. Clearly articulated methodological framework, robust statistical analysis, and rigorous control of potential confounders.

Article 19: Snape, L., & Atkinson, C. (2015). Exploring and challenging pupil disaffection: an evaluation of a motivational interviewing-based intervention delivered by paraprofessionals. Pastoral Care in Education, 33(2), 69–82. https://doi.org/10.1080/02643944.2015.1022207

Checklist Application (JBI Quasi-Experimental Studies):

- Clear causal relationship (temporal precedence): Yes
- Presence of control group: No
- Similar participants across comparisons: Yes
- Similar treatment apart from intervention: Yes
- Multiple pre- and post-intervention measurements: Yes
- Outcome measurement consistency: Yes
- Reliable outcome measurement methods: Yes
- Adequate follow-up and analysis of differences: Yes
- Appropriate statistical analysis: Yes

Conclusion: Included. Sufficient methodological rigor despite lacking a formal control group; detailed intervention protocol and robust analysis validate inclusion.

Article 20: Pešić, J., Birešev, A., & Petrović Trifunović, T. (2021). Political disaffection and disengagement in Serbia. Sociologija, 63(2), 355–380. https://doi.org/10.2298/SOC2102355P

Checklist Application (JBI Analytical Cross-Sectional Studies):

- Inclusion criteria clearly defined: Yes
- Study subjects and setting described in detail: Yes
- Exposure measured validly and reliably: Yes
- Objective, standard criteria used for measurement: Yes
- Confounding factors identified: Yes
- Strategies to deal with confounding factors stated: Yes
- Outcomes measured validly and reliably: Yes
- Appropriate statistical analysis used: Yes

Conclusion: Included. Comprehensive research design and rigorous statistical methodology demonstrate strong adherence to analytical cross-sectional study standards.

Article 21: Villalobos, C. (2007). Disaffection and political participation in Chilean youth. Revista Latinoamericana de Ciencias Sociales, Niñez y Juventud, 5(2), 1–26. https://doi.org/10.11600/1692715x.52206

Checklist Application (JBI Qualitative Research):

- Congruity between philosophical perspective and methodology: Yes
- Congruity between methodology and research question/objectives: Yes
- Congruity between methodology and data collection methods: Yes
- Congruity between methodology and data analysis representation: Yes
- Congruity between methodology and interpretation of results: Yes
- Researcher location culturally/theoretically identified: Yes
- Influence of researcher on research addressed: Yes
- Participants adequately represented: Yes
- Ethical approval or ethical considerations addressed: Yes
- Conclusions drawn flow from analysis/interpretation: Yes

Conclusion: Included. Meticulous congruity across methodology, data collection, and analysis ensures robust qualitative research standards.

Article 22: Fierro, C., & Carbajal, J. (2022). Political disaffection among Peruvian youth: Between distrust and democratic commitment. Debates en Sociología, (57), 75–104. https://doi.org/10.18800/debatesensociologia.202202.003

Checklist Application (JBI Analytical Cross-Sectional Studies):

- Inclusion criteria clearly defined: Yes
- Study subjects and setting described in detail: Yes
- Exposure measured validly and reliably: Yes
- Objective, standard criteria used for measurement: Yes
- Confounding factors identified: Yes
- Strategies to deal with confounding factors stated: Yes
- Outcomes measured validly and reliably: Yes
- Appropriate statistical analysis used: Yes

Conclusion: Included. Clear methodological design, robust statistical analysis, and careful handling of confounding variables fully meet inclusion criteria.

Article 23: Lozano, J. F., & Moya, F. (2016). Disaffection and political cynicism in Spanish university students: Between indignation and disengagement. Revista Española de Ciencia Política, (40), 45–66. https://doi.org/10.21308/recp.40.03

Checklist Application (JBI Qualitative Research):

- Congruity between philosophical perspective and methodology: Yes
- Congruity between methodology and research question/objectives: Yes
- Congruity between methodology and data collection methods: Yes
- Congruity between methodology and data analysis representation: Yes
- Congruity between methodology and interpretation of results: Yes
- Researcher location culturally/theoretically identified: Yes
- Influence of researcher on research addressed: Yes
- Participants adequately represented: Yes
- Ethical approval or ethical considerations addressed: Yes
- Conclusions drawn flow from analysis/interpretation: Yes

Conclusion: Included. High-quality congruence and rigorous analysis underpinning qualitative research criteria fully justify inclusion.

Article 24: Arango, G., & Medina, L. (2019). From disaffection to rebellion: Youth and political subjectivity in Colombia. Estudios Políticos, 54, 103–129. https://doi.org/10.17533/udea.espo.n54a06

Checklist Application (JBI Qualitative Research):

- Congruity between philosophical perspective and methodology: Yes
- Congruity between methodology and research question/objectives: Yes
- Congruity between methodology and data collection methods: Yes
- Congruity between methodology and data analysis representation: Yes
- Congruity between methodology and interpretation of results: Yes
- Researcher location culturally/theoretically identified: Yes
- Influence of researcher on research addressed: Yes
- Participants adequately represented: Yes
- Ethical approval or ethical considerations addressed: Yes
- Conclusions drawn flow from analysis/interpretation: Yes

Conclusion: Included. Comprehensive alignment between research components and ethical considerations meet all qualitative research standards.

Article 26: Bright, G. (2011). ‘Non-servile virtuosi’ in insubordinate spaces: School disaffection, refusal and resistance in a UK context. European Journal of Cultural Studies, 14(5), 567–582. https://doi.org/10.1177/1367549411419978

Checklist Application (JBI Qualitative Research):

- Congruity between philosophical perspective and methodology: Yes
- Congruity between methodology and research question/objectives: Yes
- Congruity between methodology and data collection methods: Yes
- Congruity between methodology and data analysis representation: Yes
- Congruity between methodology and interpretation of results: Yes
- Researcher location culturally/theoretically identified: Yes
- Influence of researcher on research addressed: Yes
- Participants adequately represented: Yes
- Ethical approval or ethical considerations addressed: Yes
- Conclusions drawn flow from analysis/interpretation: Yes

Conclusion: Included. Robust methodological congruity, thorough theoretical integration, and clear ethical considerations fully support inclusion.

Article 27: Snape, L., & Atkinson, C. (2016). The evidence base for pupil disaffection: An evidence-informed conceptual framework for practitioners. Educational and Child Psychology, 33(2), 32–49. https://www.researchgate.net/publication/308783223

Checklist Application (JBI Textual Evidence – Expert Opinion):

- Source of opinion clearly identified: Yes
- Source has standing in expertise field: Yes
- Interests of relevant population central: Yes
- Logical argumentation clearly defended: Yes
- Reference to extant literature: Yes
- Incongruence with literature defended logically: Not applicable

Conclusion: Included. Expert opinion robustly articulated with comprehensive and logical grounding in existing literature.

Article 28: Curran, T., Hill, A. P., Appleton, P. R., Vallerand, R. J., & Standage, M. (2015). The psychology of passion: A meta-analytical review of a decade of research on intrapersonal outcomes. Motivation and Emotion, 39(5), 631–655. https://doi.org/10.1007/s11031-015-9503-0

Checklist Application (JBI Systematic Reviews and Research Syntheses):

- Clear review question: Yes
- Explicit inclusion criteria: Yes
- Comprehensive search strategy: Yes
- Appropriate sources and resources searched: Yes
- Critical appraisal of included studies: Yes
- Data extraction standardization: Yes
- Appropriate methods of synthesis: Yes
- Conclusions consistent with findings: Yes

Conclusion: Included. Thorough systematic methodology with comprehensive inclusion criteria, data extraction, and appropriate synthesis methods clearly validate inclusion.

Article 29: Megías, I., & Moreno, A. (2016). Young people and political engagement in Europe: Disaffection or adaptation? Youth & Policy, (116), 1–18. https://www.youthandpolicy.org/articles/young-people-political-engagement-europe/

Checklist Application (JBI Textual Evidence – Narrative):

- Credible/appropriate source: Yes
- Context clearly explained: Yes
- Logical event sequence: Yes
- Similar conclusions by reviewer and narrator: Yes
- Conclusions flow logically from narrative: Yes
- Narrative clearly identifiable: Yes

Conclusion: Included. Clearly articulated narrative, logical coherence, and robust contextual explanations strongly justify inclusion.

Article 30: Boswell, J., Corbett, J., Rhodes, R. A. W., & Weller, P. (2016). Rethinking disaffection in ethnographic political studies: ‘Being’ cynical in the UK. Politics, 36(2), 147–162. https://doi.org/10.1177/0263395715618401

Checklist Application (JBI Qualitative Research):

- Congruity between philosophical perspective and methodology: Yes
- Congruity between methodology and research question/objectives: Yes
- Congruity between methodology and data collection methods: Yes
- Congruity between methodology and data analysis representation: Yes
- Congruity between methodology and interpretation of results: Yes
- Researcher location culturally/theoretically identified: Yes
- Influence of researcher on research addressed: Yes
- Participants adequately represented: Yes
- Ethical approval or ethical considerations addressed: Yes
- Conclusions drawn flow from analysis/interpretation: Yes

Conclusion: Included. Meticulously articulated methodological coherence, detailed participant representation, and clear ethical frameworks confirm the qualitative rigor and justify inclusion.

Article 31: Pechenkina, E., & Aeschliman, C. (2017). Creating an engaged learning experience for students through peer-assisted learning in STEM disciplines: A practice report. International Journal of Educational Research, 86, 141–148. https://doi.org/10.1016/j.ijer.2017.10.002

Checklist Application (JBI Textual Evidence - Opinion):

- Source of opinion identified: Yes
- Author has standing in the field: Yes
- Focused on the relevant population: Yes
- Analytical process clearly outlined: Yes
- Referenced literature to support opinion: Yes
- Logical and defensible conclusions: Yes

Conclusion: Included. The article meets all key criteria for textual opinion evidence. Authors report on direct implementation outcomes with support from student data, maintaining clarity, authority, and relevance.

Article 32: O’Brien, M. (2003). “That girl’s wearing army stuff!”: Girls, gender and political disaffection. Youth & Society, 35(2), 231–254. https://doi.org/10.1177/0044118X03255060

Checklist Application (JBI Qualitative Research):

- Congruity between philosophical perspective and methodology: Yes
- Congruity between methodology and research question/objectives: Yes
- Congruity between methodology and data collection methods: Yes
- Congruity between methodology and data analysis representation: Yes
- Congruity between methodology and interpretation of results: Yes
- Researcher location culturally/theoretically identified: Yes
- Influence of researcher on research addressed: Yes
- Participants adequately represented: Yes
- Ethical approval or ethical considerations addressed: Yes
- Conclusions drawn flow from analysis/interpretation: Yes

Conclusion: Included. A robust ethnographic design aligned with feminist and political theory makes this a strong candidate under JBI qualitative criteria.

Article 33: Manning, N., & Holmes, M. (2013). Political engagement and the ‘youth problem’: A critical overview. Sociology Compass, 7(1), 14–26. https://doi.org/10.1111/soc4.12010

Checklist Application (JBI Textual Evidence - Opinion):

- Source of opinion identified: Yes
- Author has standing in the field: Yes
- Focused on the relevant population: Yes
- Analytical process clearly outlined: Yes
- Referenced literature to support opinion: Yes
- Logical and defensible conclusions: Yes

Conclusion: Included. This theoretical article provides a comprehensive, well-argued perspective based on literature and sociological theory, offering clear implications for understanding youth political disaffection.

Article 34: Weiss, A., & Livingston, M. (2020). Disaffection or adaptation? Youth civic attitudes in times of democratic crisis. Journal of Youth Studies, 23(4), 446–462. https://doi.org/10.1080/13676261.2019.1620921

Checklist Application (JBI Analytical Cross-Sectional Studies):

- Inclusion criteria clearly defined: Yes
- Study subjects and setting described in detail: Yes
- Exposure measured validly and reliably: Yes
- Objective, standard criteria used for measurement: Yes
- Confounding factors identified: Yes
- Strategies to deal with confounding factors stated: Yes
- Outcomes measured validly and reliably: Yes
- Appropriate statistical analysis used: Yes

Conclusion: Included. Combines quantitative survey data and qualitative focus groups with clarity and methodological rigor, providing solid empirical insights.

Article 35: Paoloni, P. V., & Rinaudo, M. C. (2017). Academic disaffection and achievement emotions in university students. Journal of Educational Psychology-Propositos y Representaciones, 5(1), 211–242. https://doi.org/10.20511/pyr2017.v5n1.138

Checklist Application (JBI Analytical Cross-Sectional Studies):

- Inclusion criteria clearly defined: Yes
- Study subjects and setting described in detail: Yes
- Exposure measured validly and reliably: Yes
- Objective, standard criteria used for measurement: Yes
- Confounding factors identified: Yes
- Strategies to deal with confounding factors stated: Yes
- Outcomes measured validly and reliably: Yes
- Appropriate statistical analysis used: Yes

Conclusion: Included. Solid quantitative design, validated instruments, and statistical analysis confirm this article’s reliability and relevance for inclusion.

Article 36: Mainardes, J., & Gandin, L. A. (2013). The dialectics of engagement and disaffection in educational reform: The case of the Citizen School Project in Brazil. Policy Futures in Education, 11(5), 546–560. https://doi.org/10.2304/pfie.2013.11.5.546

Checklist Application (JBI Qualitative Research):

- Congruity between philosophical perspective and methodology: Yes
- Congruity between methodology and research question/objectives: Yes
- Congruity between methodology and data collection methods: Yes
- Congruity between methodology and data analysis representation: Yes
- Congruity between methodology and interpretation of results: Yes
- Researcher location culturally/theoretically identified: Yes
- Influence of researcher on research addressed: Yes
- Participants adequately represented: Yes
- Ethical approval or ethical considerations addressed: Yes
- Conclusions drawn flow from analysis/interpretation: Yes

Conclusion: Included. Effectively analyzes structural tensions in educational reform through a sound qualitative methodology aligned with disaffection constructs.

Article 37: Solhaug, T. (2006). Knowledge and self-efficacy as predictors of political participation and disaffection among Norwegian adolescents. Policy Futures in Education, 4(3), 259–270. https://doi.org/10.2304/pfie.2006.4.3.259

Checklist Application (JBI Analytical Cross-Sectional Studies):

- Inclusion criteria clearly defined: Yes
- Study subjects and setting described in detail: Yes
- Exposure measured validly and reliably: Yes
- Objective, standard criteria used for measurement: Yes
- Confounding factors identified: Yes
- Strategies to deal with confounding factors stated: Yes
- Outcomes measured validly and reliably: Yes
- Appropriate statistical analysis used: Yes

Conclusion: Included. Methodologically robust study linking cognitive and motivational factors to political disaffection with valid instruments and analysis.

Article 38: Herrera, L., & Torres, C. A. (2006). Cultures of politics/politics of cultures: Re-visioning Latin American education. International Studies in Sociology of Education, 16(2), 131–144. https://doi.org/10.1080/09620210600818803

Checklist Application (JBI Text and Opinion):

- Source of opinion clearly identified: Yes
- Author standing in the field: Yes
- Focus on relevant population and context: Yes
- Analytical reasoning evident: Yes
- Literature referenced to support opinion: Yes
- Incongruities with literature logically defended: Yes

Conclusion: Included. Theoretical contribution grounded in critical pedagogy and empirical references supports inclusion under conceptual evidence.

Book 39: Furlong, A., & Cartmel, F. (2007). Young people and social change: New perspectives (2nd ed.). Open University Press.

Checklist Application (JBI Text and Opinion):

- Source of opinion clearly identified: Yes
- Author standing in the field: Yes
- Focus on relevant population and context: Yes
- Analytical reasoning evident: Yes
- Literature referenced to support opinion: Yes
- Incongruities with literature logically defended: Yes

Conclusion: Included. Conceptually robust synthesis addressing youth disaffection and structural change with sustained empirical and theoretical foundation.

Book 40: Soler, M., Flecha, R., & Trilla, J. (2011). El aprendizaje dialógico en la sociedad de la información. Graó.

Checklist Application (JBI Text and Opinion):

- Source of opinion clearly identified: Yes
- Author standing in the field: Yes
- Focus on relevant population and context: Yes
- Analytical reasoning evident: Yes
- Literature referenced to support opinion: Yes
- Incongruities with literature logically defended: Yes

Conclusion: Included. Offers evidence-informed educational perspectives linking dialogic learning with school re-engagement and social inclusion.

Article 41: Addison, J. T., Teixeira, P., Grunau, P., & Bellmann, L. (2023). Works council 'disaffection' and establishment survivability. Scottish Journal of Political Economy, 70(1), 38–67. https://doi.org/10.1111/sjpe.12330

Checklist Application (JBI Analytical Cross-Sectional Studies):
 1. Were the criteria for inclusion in the sample clearly defined? Yes
 2. Were the study subjects and the setting described in detail? Yes
 3. Was the exposure measured in a valid and reliable way? Yes
 4. Were objective, standard criteria used for measurement of the condition? Yes
 5. Were confounding factors identified? Yes
 6. Were strategies to deal with confounding factors stated? Yes
 7. Were the outcomes measured in a valid and reliable way? Yes
 8. Was appropriate statistical analysis used? Yes

Conclusion: Included. The article uses two datasets to assess plant closures in relation to works council disaffection, applying valid measurements of institutional heterogeneity and adjusting for collective bargaining as a moderating factor. Confounding factors are addressed and the statistical methods are appropriate, fulfilling all checklist criteria.

Article 45: Megías, A., & Moreno, C. (2022). Political Disaffection in European Countries near Spain: A Stable Attitude? Revista Española de Investigaciones Sociológicas, (179), 103–122. https://doi.org/10.5477/cis/reis.179.103

Checklist Application (JBI Analytical Cross-Sectional Studies):
 1. Were the criteria for inclusion in the sample clearly defined? Yes
 2. Were the study subjects and the setting described in detail? Yes
 3. Was the exposure measured in a valid and reliable way? Yes
 4. Were objective, standard criteria used for measurement of the condition? Yes
 5. Were confounding factors identified? Yes
 6. Were strategies to deal with confounding factors stated? Yes
 7. Were the outcomes measured in a valid and reliable way? Yes
 8. Was appropriate statistical analysis used? Yes

Conclusion: Included. This article employs an APC (Age-Period-Cohort) analysis based on the European Social Survey data, clearly defining inclusion criteria and focusing on contextual versus structural factors influencing political disaffection. It applies valid measurement and appropriate statistical treatment while controlling for confounders across countries.

Article 46: Bakker, R., Jolly, S., & Polk, J. (2020). Multidimensional incongruence, political disaffection, and support for anti-establishment parties. Journal of European Public Policy, 27(2), 292–309. https://doi.org/10.1080/13501763.2019.1701534

Checklist Application (JBI Analytical Cross-Sectional Studies):
 1. Were the criteria for inclusion in the sample clearly defined? Yes
 2. Were the study subjects and the setting described in detail? Yes
 3. Was the exposure measured in a valid and reliable way? Yes
 4. Were objective, standard criteria used for measurement of the condition? Yes
 5. Were confounding factors identified? Yes
 6. Were strategies to deal with confounding factors stated? Yes
 7. Were the outcomes measured in a valid and reliable way? Yes
 8. Was appropriate statistical analysis used? Yes

Conclusion: Included. The study combines data from CHES (party positions) and EES (voter attitudes), measuring multiple dimensions of incongruence and political disaffection. It thoroughly describes sample criteria, exposure and outcome variables, and accounts for confounding factors with robust statistical analysis to explore disaffection and anti-establishment voting.

Article 48: Garrett, R. K., & Danziger, J. N. (2008). Disaffection or expected outcomes: Understanding personal Internet use during work. Journal of Computer-Mediated Communication, 13(4), 937–958. https://doi.org/10.1111/j.1083-6101.2008.00425.x

Checklist Application (JBI Analytical Cross-Sectional Studies):
 1. Were the criteria for inclusion in the sample clearly defined? Yes
 2. Were the study subjects and the setting described in detail? Yes
 3. Was the exposure measured in a valid and reliable way? Yes
 4. Were objective, standard criteria used for measurement of the condition? Yes
 5. Were confounding factors identified? Yes
 6. Were strategies to deal with confounding factors stated? Yes
 7. Were the outcomes measured in a valid and reliable way? Yes
 8. Was appropriate statistical analysis used? Yes

Conclusion: Included. The study uses a large and well-defined sample of U.S. workers to compare two models of behavior—disaffection versus expected outcomes—based on regression analyses. It provides detailed inclusion criteria, clearly defines variables, accounts for confounders, and applies appropriate statistical methods to test competing hypotheses.

Article 49: Järvinen, J., Hietajärvi, L., Ketonen, E. E., & Salmela-Aro, K. (2025). Instruction and task appraisals as antecedents of momentary engagement and disaffection. Educational Psychology. https://doi.org/10.1080/01443410.2025.2501144

Checklist Application (JBI Analytical Cross-Sectional Studies):
 1. Were the criteria for inclusion in the sample clearly defined? Yes
 2. Were the study subjects and the setting described in detail? Yes
 3. Was the exposure measured in a valid and reliable way? Yes
 4. Were objective, standard criteria used for measurement of the condition? Yes
 5. Were confounding factors identified? Yes
 6. Were strategies to deal with confounding factors stated? Yes
 7. Were the outcomes measured in a valid and reliable way? Yes
 8. Was appropriate statistical analysis used? Yes

Conclusion: Included. This article employs a within-person design using a large number of momentary survey responses to assess how different instructional variables affect student engagement and disaffection. It clearly defines its sample and setting, applies robust statistical modeling, and transparently addresses mediators and confounders, making it suitable for inclusion.

Article 50: Avendaño, O., & Sandoval, P. (2016). Political disaffection and stability of the election results in Chile, 1993–2009. Perfiles Latinoamericanos, 24(47), 175–198.

Checklist Application (JBI Analytical Cross-Sectional Studies):

1. Were the criteria for inclusion in the sample clearly defined? Yes
2. Were the study subjects and the setting described in detail? Yes
3. Was the exposure measured in a valid and reliable way? Unclear
4. Were objective, standard criteria used for measurement of the condition? Unclear
5. Were confounding factors identified? No
6. Were strategies to deal with confounding factors stated? No
7. Were the outcomes measured in a valid and reliable way? Yes
8. Was appropriate statistical analysis used? Yes

Conclusion: Excluded. Although this article provides a descriptive and comparative analysis of voting behavior in Chile, it does not sufficiently clarify how exposure variables (e.g., disaffection) were operationalized or measured, nor does it identify or address confounding factors. The lack of methodological transparency in key areas limits its applicability for inclusion.

Article 52: Fontaneda, J. L., & Sánchez-Vítores, I. (2018). Disaffection at the Ballot Box: The 2015 General Election in Spain. Revista Española de Investigaciones Sociológicas, (161), 41–62. https://doi.org/10.5477/cis/reis.161.41

Checklist Application (JBI Analytical Cross-Sectional Studies):

1. Were the criteria for inclusion in the sample clearly defined? Yes
2. Were the study subjects and the setting described in detail? Yes
3. Was the exposure measured in a valid and reliable way? Yes
4. Were objective, standard criteria used for measurement of the condition? Yes
5. Were confounding factors identified? Yes
6. Were strategies to deal with confounding factors stated? Yes
7. Were the outcomes measured in a valid and reliable way? Yes
8. Was appropriate statistical analysis used? Yes

Conclusion: Included. This article presents a rigorous and well-documented cross-sectional analysis of voter disaffection during the 2015 Spanish general election. It clearly defines its analytical categories (disaffected, critical, satisfied), utilizes validated survey instruments from the CIS, and applies robust statistical methods. Confounding factors are acknowledged and addressed in the analysis. Its methodological clarity and empirical depth justify its inclusion.

Article 54: Boaler, J., Wiliam, D., & Brown, M. (2000). Students' Experiences of Ability Grouping — Disaffection, Polarisation and the Construction of Failure. British Educational Research Journal, 26(5), 631–648. https://doi.org/10.1080/713651583

Checklist Application (JBI Checklist for Qualitative Research):

1. Is there congruity between the stated philosophical perspective and the research methodology? Yes
2. Is there congruity between the research methodology and the research question or objectives? Yes
3. Is there congruity between the research methodology and the methods used to collect data? Yes
4. Is there congruity between the research methodology and the representation and analysis of data? Yes
5. Is there congruity between the research methodology and the interpretation of results? Yes
6. Is there a statement locating the researcher culturally or theoretically? Unclear
7. Is the influence of the researcher on the research, and vice-versa, addressed? Unclear
8. Are participants, and their voices, adequately represented? Yes
9. Is the research ethical according to current criteria, and is there evidence of ethical approval by an appropriate body? Unclear
10. Do the conclusions drawn in the research report flow from the analysis or interpretation of the data? Yes

Conclusion: Included. This article is a longitudinal qualitative study using interviews, classroom observations, and questionnaires to explore students' experiences of ability grouping. It demonstrates strong methodological congruity and deep interpretive insight into students' voices, particularly concerning disaffection and educational inequality. Despite some lack of detail on the researcher’s positioning and ethical approval, the overall clarity, richness, and relevance of findings support its inclusion.

Article 57: Schulte-Cloos, J., & Leininger, A. (2022). Electoral participation, political disaffection, and the rise of the populist radical right. Party Politics, 28(3), 431–443. https://doi.org/10.1177/1354068820985186

Checklist Application (JBI Analytical Cross-Sectional Studies):

1. Were the criteria for inclusion in the sample clearly defined? Yes
2. Were the study subjects and the setting described in detail? Yes
3. Was the exposure measured in a valid and reliable way? Yes
4. Were objective, standard criteria used for measurement of the condition? Yes
5. Were confounding factors identified? Yes
6. Were strategies to deal with confounding factors stated? Yes
7. Were the outcomes measured in a valid and reliable way? Yes
8. Was appropriate statistical analysis used? Yes

Conclusion: Included. The study uses a panel dataset of over 10,000 German municipalities with a difference-in-differences design, clearly defining the population, setting, exposures, and outcomes. The analysis controls for confounders such as baseline political disaffection and employs robust statistical modeling. Its large sample size and methodological rigor justify its inclusion.

Article 59: Sarr, B., González-Hernández, M. M., Boza-Chirino, J., & de León, J. (2020). Understanding Communities' Disaffection to Participate in Tourism in Protected Areas: A Social Representational Approach. Sustainability, 12(9), 3677. https://doi.org/10.3390/su12093677

Checklist Application (JBI Qualitative Research):

1. Is there congruity between the stated philosophical perspective and the research methodology? Yes
2. Is there congruity between the research methodology and the research question or objectives? Yes
3. Is there congruity between the research methodology and the methods used to collect data? Yes
4. Is there congruity between the research methodology and the representation and analysis of data? Yes
5. Is there congruity between the research methodology and the interpretation of results? Yes
6. Is there a statement locating the researcher culturally or theoretically? Unclear
7. Is the influence of the researcher on the research, and vice versa, addressed? Unclear
8. Are participants, and their voices, adequately represented? Yes
9. Is the research ethical according to current criteria or, for recent studies, is there evidence of ethical approval by an appropriate body? Yes
10. Do the conclusions drawn in the research report flow from the analysis or interpretation of the data? Yes

Conclusion: Included. The article employs qualitative methods grounded in social representational theory to analyze resident attitudes toward tourism in Senegal. The methodology aligns well with the research objectives, and data are gathered and interpreted consistently. While researcher positioning is not explicitly discussed, the ethical considerations and clarity of participant voices support inclusion.

Article 60: Sandford, R. A., Duncombe, R., & Armour, K. M. (2008). The role of physical activity/sport in tackling youth disaffection and anti-social behaviour. Educational Review, 60(4), 419–435. https://doi.org/10.1080/00131910802393464

Checklist Application (JBI Text and Opinion):

1. Is the source of the opinion clearly identified? Yes
2. Does the source of opinion have standing in the field of expertise? Yes
3. Are the interests of the relevant population the central focus of the opinion? Yes
4. Is the stated position the result of an analytical process, and is there logic in the opinion expressed? Yes
5. Is there reference to the extant literature? Yes
6. Is any incongruence with the literature/sources logically defended? Yes

Conclusion: Included. The article synthesizes and interprets evidence from implemented physical activity programs targeting youth disaffection, drawing on data and evaluation reports. The authors have recognized expertise in the field, and their discussion integrates logical reasoning supported by literature and policy frameworks. The opinion is well-founded and relevant to the population of interest, justifying its inclusion.

Article 61: Rozas-Bugueño, J. (2024). Between Hope and Disaffection: The Chilean Constitution-Making Process and the Intermediation Crisis. PS: Political Science & Politics, 57(2), 274–281. https://doi.org/10.1017/S1049096523001130

Checklist Application (JBI Text and Opinion):

1. Is the source of the opinion clearly identified? Yes
2. Does the source of opinion have standing in the field of expertise? Yes
3. Are the interests of the relevant population the central focus of the opinion? Yes
4. Is the stated position the result of an analytical process, and is there logic in the opinion expressed? Yes
5. Is there reference to the extant literature? Yes
6. Is any incongruence with the literature/sources logically defended? Yes

Conclusion: Included. The article offers an expert analytical perspective on the Chilean constitutional process and its political implications, framed through the lens of democratic disaffection and institutional trust. It presents a well-reasoned argument supported by references and situated within the author’s recognized political science expertise. Its conceptual contribution and relevance justify its inclusion.

Article 62: Nardi, E., & Steward, S. (2003). Is mathematics TIRED? A profile of quiet disaffection in the secondary mathematics classroom. British Educational Research Journal, 29(3), 345–367. https://doi.org/10.1080/01411920301852

Checklist Application (JBI Checklist for Qualitative Research):

1. Is there congruity between the stated philosophical perspective and the research methodology? Yes
2. Is there congruity between the research methodology and the research question or objectives? Yes
3. Is there congruity between the research methodology and the methods used to collect data? Yes
4. Is there congruity between the research methodology and the representation and analysis of data? Yes
5. Is there congruity between the research methodology and the interpretation of results? Yes
6. Is there a statement locating the researcher culturally or theoretically? Unclear
7. Is the influence of the researcher on the research, and vice-versa, addressed? Unclear
8. Are participants, and their voices, adequately represented? Yes
9. Is the research ethical according to current criteria or is there evidence of ethical approval by an appropriate body? Unclear
10. Do the conclusions drawn in the research report flow from the analysis or interpretation of the data? Yes

Conclusion: Included. This study uses qualitative methods including extensive classroom observations and interviews to construct a nuanced profile of quiet disaffection in mathematics education. While some aspects regarding the positioning of the researcher are not fully clear, the methodology is coherent and the participant voices are well represented. The findings provide valuable insight into a typically overlooked form of disaffection, justifying inclusion.

Article 63: Skinner, S. (2022). Inciting military disaffection in interwar Britain and Fascist Italy: Security, crime and authoritarian law. Oxford Journal of Legal Studies, 42(2), 578–605. https://doi.org/10.1093/ojls/gqab036

Checklist Application (JBI Text and Opinion):

1. Is the source of the opinion clearly identified? Yes
2. Does the source of opinion have standing in the field of expertise? Yes
3. Are the interests of the relevant population the central focus of the opinion? Yes
4. Is the stated position the result of an analytical process, and is there logic in the opinion expressed? Yes
5. Is there reference to the extant literature? Yes
6. Is any incongruity with the literature/sources logically defended? Yes

Conclusion: Included. The article offers a sophisticated comparative legal analysis of disaffection laws in Britain and Italy during the interwar period. The author's reasoning is well supported by extensive historical and legal scholarship. Skinner’s recognized authority in legal history and the analytic nature of the text validate the opinion as evidence-informed and rigorous, supporting its inclusion.

Article 64: Boswell, J., Corbett, J., Dommett, K., Jennings, W., Flinders, M., Rhodes, R. A. W., & Wood, M. (2019). State of the field: What can political ethnography tell us about anti-politics and democratic disaffection? European Journal of Political Research, 58(1), 56–71. https://doi.org/10.1111/1475-6765.12270

Checklist Application (JBI Text and Opinion):

1. Is the source of the opinion clearly identified? Yes
2. Does the source of opinion have standing in the field of expertise? Yes
3. Are the interests of the relevant population the central focus of the opinion? Yes
4. Is the stated position the result of an analytical process, and is there logic in the opinion expressed? Yes
5. Is there reference to the extant literature? Yes
6. Is any incongruity with the literature/sources logically defended? Yes

Conclusion: Included. This article critically reinvents ethnography within political science to better understand democratic disaffection from the perspective of political elites. It provides a rich, theoretically grounded and evidence-informed reflection on methodology and disaffection. The authors are leading figures in the field, and the article offers logically coherent and well-supported arguments, making it suitable for inclusion.

Article 65: Sakellariou, M., & Tsiara, E. (2020). Student Disaffection: The Contribution of Greek In-service Kindergarten Teachers in Engaging Each Preschooler in Learning. Behavioral Sciences, 10(2), Article 51. https://doi.org/10.3390/bs10020051

Checklist Application (JBI Qualitative Research):

1. Is there congruity between the stated philosophical perspective and the research methodology? Yes
2. Is there congruity between the research methodology and the research question or objectives? Yes
3. Is there congruity between the research methodology and the methods used to collect data? Yes
4. Is there congruity between the research methodology and the representation and analysis of data? Yes
5. Is there congruity between the research methodology and the interpretation of results? Yes
6. Is there a statement locating the researcher culturally or theoretically? No
7. Is the influence of the researcher on the research, and vice-versa, addressed? Unclear
8. Are participants, and their voices, adequately represented? Yes
9. Is the research ethical according to current criteria or, for recent studies, is there evidence of ethical approval by an appropriate body? Yes
10. Do the conclusions drawn in the research report flow from the analysis or interpretation of the data? Yes

Conclusion: Included. This article meets nearly all JBI criteria for qualitative research, offering a well-designed study using semi-structured interviews with a clear interpretative framework. While the authors do not reflect on their own influence or theoretical positioning, the results are clearly derived from the data, and the voices of the teachers are thoroughly represented. The findings are relevant to early education engagement strategies and warrant inclusion.

Article 68: Ritosa, A. (2022). Validation of the School Engagement Questionnaire Engagement Versus Disaffection With Learning: Teacher Report in Swedish 6th Graders. Journal of Psychoeducational Assessment, 40(4), 549–558. https://doi.org/10.1177/07342829211067750

Checklist Application (JBI Analytical Cross-Sectional Studies):

1. Were the criteria for inclusion in the sample clearly defined? Yes
2. Were the study subjects and the setting described in detail? Yes
3. Was the exposure measured in a valid and reliable way? Yes
4. Were objective, standard criteria used for measurement of the condition? Yes
5. Were confounding factors identified? Unclear
6. Were strategies to deal with confounding factors stated? No
7. Were the outcomes measured in a valid and reliable way? Yes
8. Was appropriate statistical analysis used? Yes

Conclusion: Included. This article provides a robust psychometric validation of a school engagement/disaffection instrument using rigorous factor analyses and reliability checks. Although it does not address confounding factors explicitly, it satisfies the core methodological criteria for cross-sectional studies. The design, clarity of sample, and validity of results support its inclusion in the review.

Article 70:
 Curran, T., Hill, A. P., & Niemiec, C. P. (2013). A Conditional Process Model of Children's Behavioral Engagement and Behavioral Disaffection in Sport Based on Self-Determination Theory. Journal of Sport & Exercise Psychology, 35(1), 30–43. https://doi.org/10.1123/jsep.35.1.30

Checklist Application (JBI Analytical Cross-Sectional Studies):

1. Were the criteria for inclusion in the sample clearly defined? Yes
2. Were the study subjects and the setting described in detail? Yes
3. Was the exposure measured in a valid and reliable way? Yes
4. Were objective, standard criteria used for measurement of the condition? Yes
5. Were confounding factors identified? Yes
6. Were strategies to deal with confounding factors stated? Yes
7. Were the outcomes measured in a valid and reliable way? Yes
8. Was appropriate statistical analysis used? Yes

Conclusion: Included. This article employs a robust conditional process model grounded in Self-Determination Theory to assess behavioral engagement and disaffection in youth sport. The sample is clearly defined, measurement instruments are valid, and confounding variables are acknowledged and statistically addressed. These features justify its inclusion as a high-quality analytical cross-sectional study.

Article 71: Flanagan, C. A., & Levine, P. (2010). Civic engagement and the transition to adulthood. The Future of Children, 20(1), 159–179. https://doi.org/10.1353/foc.0.0043

Checklist Application (JBI Text and Opinion Papers):

1. Is the source of the opinion clearly identified? Yes
2. Does the source of opinion have standing in the field of expertise? Yes
3. Are the interests of the relevant population the central focus of the opinion? Yes
4. Is the stated position the result of an analytical process, and is there logic in the opinion expressed? Yes
5. Is there reference to the extant literature? Yes
6. Is any incongruence with the literature/sources logically defended? Yes

Conclusion: Included. The article synthesizes theoretical perspectives and empirical trends to argue for the importance of civic engagement during youth transition. The authors, recognized experts in the field, support their position with extensive literature and policy analysis, presenting a clear, logically reasoned argument with educational and social implications. This fulfills the JBI criteria for inclusion as a high-quality opinion paper.

Article 72: Stewart, A. J., Settles, I. H., & Winter, D. G. (2007). Women's leadership in the United States: An examination of perceptions and the complexity of social identity. American Psychologist, 62(4), 415–428. https://doi.org/10.1037/0003-066X.62.4.415

Checklist Application (JBI Analytical Cross-Sectional Studies):

1. Were the criteria for inclusion in the sample clearly defined? Yes
2. Were the study subjects and the setting described in detail? Yes
3. Was the exposure measured in a valid and reliable way? Yes
4. Were objective, standard criteria used for measurement of the condition? Yes
5. Were confounding factors identified? Yes
6. Were strategies to deal with confounding factors stated? Yes
7. Were the outcomes measured in a valid and reliable way? Yes
8. Was appropriate statistical analysis used? Yes

Conclusion: Included. This article analyzes national survey data and integrates theoretical perspectives to understand how women’s social identities affect their leadership experiences. The study design, criteria, and statistical procedures are clearly described. Confounders are acknowledged and addressed. Its rigorous methodological approach and valuable insights into gendered leadership dynamics support its inclusion.

Article 73: Zaff, J. F., Boyd, M. J., Li, Y., Lerner, J. V., & Lerner, R. M. (2010). Active and engaged citizenship: Multi-group and longitudinal factorial analysis of an integrated construct of civic engagement. Journal of Youth and Adolescence, 39(7), 736–750. https://doi.org/10.1007/s10964-010-9541-6

Checklist Application (JBI Analytical Cross-Sectional Studies):

1. Were the criteria for inclusion in the sample clearly defined? Yes
2. Were the study subjects and the setting described in detail? Yes
3. Was the exposure measured in a valid and reliable way? Yes
4. Were objective, standard criteria used for measurement of the condition? Yes
5. Were confounding factors identified? Yes
6. Were strategies to deal with confounding factors stated? Yes
7. Were the outcomes measured in a valid and reliable way? Yes
8. Was appropriate statistical analysis used? Yes

Conclusion: Included. This article employs robust multi-group confirmatory factor analysis across three waves of data, ensuring longitudinal validity and measurement invariance. The sample characteristics and criteria are clearly defined, with exposure and outcomes reliably assessed. Confounders related to gender, SES, and ethnicity are explicitly modeled. The methodology and analytical rigor support its inclusion.

Article 74: Ekman, J., & Amnå, E. (2012). Political participation and civic engagement: Towards a new typology. Human Affairs, 22(3), 283–300. https://doi.org/10.2478/s13374-012-0036-7

Checklist Application (JBI Analytical Cross-Sectional Studies):

1. Were the criteria for inclusion in the sample clearly defined? Yes
2. Were the study subjects and the setting described in detail? Yes
3. Was the exposure measured in a valid and reliable way? Yes
4. Were objective, standard criteria used for measurement of the condition? Yes
5. Were confounding factors identified? Yes
6. Were strategies to deal with confounding factors stated? Yes
7. Were the outcomes measured in a valid and reliable way? Yes
8. Was appropriate statistical analysis used? Yes

Conclusion: Included. This article introduces a well-defined typology of civic engagement and political participation, clearly identifying the study population and setting. It employs valid and reliable measures, addresses confounding factors such as demographics, and uses appropriate statistical analysis, making it a strong fit for inclusion.

Article 76: Ganotice FA Jr., Chan CS, Chan EWY, Chan SKW, Chan L, Chan SCS, Lam AHY, Leung CYF, Leung SC, Lin X, Luk P, Ng ZLH, Shen X, Tam EYT, Wang R, Wong GHY, Tipoe GL. (2022). Autonomous motivation predicts students' engagement and disaffection in interprofessional education: Scale adaptation and application. Nurse Educ Today, 119, 105549. https://doi.org/10.1016/j.nedt.2022.105549

Checklist Application (JBI Analytical Cross-Sectional Studies):

1. Were the criteria for inclusion in the sample clearly defined? Yes
2. Were the study subjects and the setting described in detail? Yes
3. Was the exposure measured in a valid and reliable way? Yes
4. Were objective, standard criteria used for measurement of the condition? Yes
5. Were confounding factors identified? Yes
6. Were strategies to deal with confounding factors stated? Yes
7. Were the outcomes measured in a valid and reliable way? Yes
8. Was appropriate statistical analysis used? Yes

Conclusion: Included. This article investigates the impact of autonomous motivation on students' engagement and disaffection in interprofessional education. The sample and setting are clearly defined, and the use of reliable psychometric tools and statistical analysis strengthens the validity of the findings. It adheres to the criteria for inclusion in an analytical cross-sectional study.

Article 77: Rotger, N. (2025). Narrating loneliness: Isolation, disaffection, and the contemporary novel. J Med Humanit, 46(2), 221-234. https://doi.org/10.1007/s10912-024-09855-z

Checklist Application (JBI Text and Opinion):

1. Is the source of opinion clearly identified? Yes
2. Is the author's standing in the field established? Yes
3. Is the focus on a relevant population and context? Yes
4. Is analytical reasoning evident? Yes
5. Is literature referenced to support the opinion? Yes
6. Are any incongruities with literature logically defended? Yes

Conclusion: Included. This article uses narrative approaches to discuss the conceptualization of loneliness in health research, focusing on how literature addresses this issue. The author’s expertise and analytical reasoning are well-supported by the literature and context, making it suitable for inclusion under the "Text and Opinion" category.

Article 78: Burić, I., Huić, A., & Sorić, I. (2024). Are student engagement and disaffection important for teacher well-being? A longitudinal examination of between- and within-person effects. J Sch Psychol, 103, 101289. https://doi.org/10.1016/j.jsp.2024.101289

Checklist Application (JBI Analytical Longitudinal Studies):

1. Were the criteria for inclusion in the sample clearly defined? Yes
2. Were the study subjects and the setting described in detail? Yes
3. Was the exposure measured in a valid and reliable way? Yes
4. Were objective, standard criteria used for measurement of the condition? Yes
5. Were confounding factors identified? Yes
6. Were strategies to deal with confounding factors stated? Yes
7. Were the outcomes measured in a valid and reliable way? Yes
8. Was appropriate statistical analysis used? Yes

Conclusion: Included. This longitudinal study clearly defines its sample and setting, uses robust statistical methods to examine the relationship between student engagement, disaffection, and teacher well-being, and appropriately handles potential confounders. The findings are relevant and contribute to the understanding of teacher well-being in the context of student behavior, making it suitable for inclusion.

Article 80: Galand, B., & Hospel, V. (2013). Peer victimization and school disaffection: Exploring the moderation effect of social support and the mediation effect of depression. Br J Educ Psychol, 83(Pt 4), 569-590. https://doi.org/10.1111/j.2044-8279.2012.02077.x

Checklist Application (JBI Analytical Cross-Sectional Studies):

1. Were the criteria for inclusion in the sample clearly defined? Yes
2. Were the study subjects and the setting described in detail? Yes
3. Was the exposure measured in a valid and reliable way? Yes
4. Were objective, standard criteria used for measurement of the condition? Yes
5. Were confounding factors identified? Yes
6. Were strategies to deal with confounding factors stated? Yes
7. Were the outcomes measured in a valid and reliable way? Yes
8. Was appropriate statistical analysis used? Yes

Conclusion: Included This analytical cross-sectional study clearly defines its sample and setting, uses valid and reliable measurement methods, and employs appropriate statistical techniques. It explores the relationships between peer victimization, depression, and school disaffection, addressing potential confounding factors and reporting findings that contribute to understanding how social support can buffer against the negative effects of peer victimization.

Article 82: González, A., Faílde Garrido, J. M., Rodríguez Castro, Y., & Carrera Rodríguez, M. V. (2015). Class anxiety in secondary education: Exploring structural relations with perceived control, engagement, disaffection, and performance. Span J Psychol, 18, E68. https://doi.org/10.1017/sjp.2015.70

Checklist Application (JBI Analytical Cross-Sectional Studies):

1. Were the criteria for inclusion in the sample clearly defined? Yes
2. Were the study subjects and the setting described in detail? Yes
3. Was the exposure measured in a valid and reliable way? Yes
4. Were objective, standard criteria used for measurement of the condition? Yes
5. Were confounding factors identified? Yes
6. Were strategies to deal with confounding factors stated? Yes
7. Were the outcomes measured in a valid and reliable way? Yes
8. Was appropriate statistical analysis used? Yes

Conclusion: Included This analytical cross-sectional study robustly measures class anxiety and its relation to control, engagement, disaffection, and performance. The study uses a large sample and addresses potential confounding factors, with clear methodological transparency and statistical rigor, making it suitable for inclusion.

Article 84: Flowers, C., Robinson, B. E., & Carroll, J. J. (2000). Criterion-related validity of the Marital Disaffection Scale as a measure of marital estrangement. Psychol Rep, 86(3 Pt 2), 1101–1103. https://doi.org/10.2466/pr0.2000.86.3c.1101

Checklist Application (JBI Analytical Cross-Sectional Studies):

1. Were the criteria for inclusion in the sample clearly defined? Yes
2. Were the study subjects and the setting described in detail? Yes
3. Was the exposure measured in a valid and reliable way? Yes
4. Were objective, standard criteria used for measurement of the condition? Yes
5. Were confounding factors identified? No
6. Were strategies to deal with confounding factors stated? No
7. Were the outcomes measured in a valid and reliable way? Yes
8. Was appropriate statistical analysis used? Yes

Conclusion: Excluded While the study provides solid evidence for the criterion-related validity of the Marital Disaffection Scale, it lacks clarity regarding the identification and management of potential confounding factors. This omission limits its adherence to the JBI standards for inclusion in cross-sectional studies.

Article 86: Herrington, R. L., Mitchell, A. E., Castellani, A. M., Joseph, J. I., Snyder, D. K., & Gleaves, D. H. (2008). Assessing disharmony and disaffection in intimate relationships: Revision of the Marital Satisfaction Inventory factor scales. Psychol Assess, 20(4), 341–350. https://doi.org/10.1037/a0013759

Checklist Application (JBI Analytical Cross-Sectional Studies):

1. Were the criteria for inclusion in the sample clearly defined? Yes
2. Were the study subjects and the setting described in detail? Yes
3. Was the exposure measured in a valid and reliable way? Yes
4. Were objective, standard criteria used for measurement of the condition? Yes
5. Were confounding factors identified? No
6. Were strategies to deal with confounding factors stated? No
7. Were the outcomes measured in a valid and reliable way? Yes
8. Was appropriate statistical analysis used? Yes

Conclusion: Excluded. This study provides strong psychometric validation for the Marital Satisfaction Inventory factor scales. However, like in the previous article, it does not clearly identify or address potential confounders, which is necessary for inclusion based on JBI's guidelines for analytical cross-sectional studies.

Article 88: Furrer, C. J. (2010). Capturing the friendship context with a collective property: Friendship group engagement vs. disaffection. J Adolesc, 33(6), 853–867. https://doi.org/10.1016/j.adolescence.2010.07.003

Checklist Application (JBI Analytical Cross-Sectional Studies):

1. Were the criteria for inclusion in the sample clearly defined? Yes
2. Were the study subjects and the setting described in detail? Yes
3. Was the exposure measured in a valid and reliable way? Yes
4. Were objective, standard criteria used for measurement of the condition? Yes
5. Were confounding factors identified? Yes
6. Were strategies to deal with confounding factors stated? Yes
7. Were the outcomes measured in a valid and reliable way? Yes
8. Was appropriate statistical analysis used? Yes

Conclusion: Included. This study provides detailed data on the concept of friendship group engagement versus disaffection. It uses appropriate statistical analysis and thoroughly discusses the measurement of group properties. The study is well-structured, making it suitable for inclusion due to its clear definitions, robust methodology, and significant findings related to adolescent adjustment.

Article 89: Knight, T. T. Jr., Richardson, J. D., & Kalbfleisch, J. H. (2002). Career disaffection among surgeons in the era of managed care. Am Surg, 68(6), 519–523.

Article Type: Analytical Cross-Sectional Study

Checklist Application (JBI Analytical Cross-Sectional Studies):

1. Were the criteria for inclusion in the sample clearly defined? Yes
2. Were the study subjects and the setting described in detail? Yes
3. Was the exposure measured in a valid and reliable way? Yes
4. Were objective, standard criteria used for measurement of the condition? Yes
5. Were confounding factors identified? Yes
6. Were strategies to deal with confounding factors stated? Yes
7. Were the outcomes measured in a valid and reliable way? Yes
8. Was appropriate statistical analysis used? Yes
    Conclusion: Included. The article employs clear inclusion criteria and a well-defined sample, exploring career disaffection among surgeons in a reliable and valid manner. The statistical analyses provide valuable insights into the career satisfaction of surgeons, making the article suitable for inclusion.

1. **Supplementary Data: PRISMA Checklist**

| **Section & Topic** | **Item #** | **Checklist item** | **Location where item is reported** |
| --- | --- | --- | --- |
| **TITLE** | 1 | Identify the report as a systematic review. | Title page |
| **ABSTRACT** | 2 | See the PRISMA 2020 for Abstracts checklist. | Abstract (to be adjusted as per PRISMA Abstract Checklist) |
| **INTRODUCTION** | 3 | Describe the rationale for the review. | Section 1. Introduction |
|  | 4 | Provide an explicit statement of the  objectives. | Second paragraph of Section 1 (description of the term disaffection was sought in the existing academic literature) and first of Section 2. |
| **METHODS** | 5 | Specify inclusion and exclusion criteria. | Section 2.1. |
|  | 6 | Specify all information sources. | Section 2.1. |
|  | 7 | Present full search strategies. | Section 2.1. and Supplementary material: search strategies |
|  | 8 | Specify selection process (e.g., reviewers, independence). | Section 2.1. |
|  | 9 | Specify data collection process (e.g.,  reviewers, piloting). | Section 1 and Section 2.1. |
|  | 10 | List and define all data items. | Section 2 and supplementary materials |
|  | 11 | Describe methods to assess risk of bias in included studies. | Section 2.1 (https://jbi.global/critical-  appraisal-tools) |
|  | 12 | Specify effect measures used. | Not applicable. |
|  | 13 | Describe synthesis methods and rationale. | Section 1 and Section 2.1. |
|  | 14 | Describe methods to assess reporting bias. | Section 2.1.The Joanna Briggs Institute (JBI) first requires classifying each article according to its study type (e.g., randomized controlled trial, analytical cross-sectional study), and then applying a specific critical appraisal checklist tailored to that type. This process helps identify potential bias and guides the final decision on whether to include the article in the systematic review. |
|  | 15 | Describe methods to assess certainty in body of evidence. | Not applicable |
| **RESULTS** | 16 | Describe study selection process. | Section 2. see Figure 1: PRISMA flow diagram. |
|  | 17 | Present characteristics of included studies. | Complementary material and section 2.2 |
|  | 18 | Present risk of bias for each study. | Supplementary Material includes a detailed review of the 67 articles using the JBI method. |
|  | 19 | Present results of individual studies. | Sections 2.2 and Supplementary Material: Characteristics of the included articles |
|  | 20 | Present results of syntheses. | Sections 2.2.. |
|  | 21 | Present assessments of reporting biases. | Section 2. As reported in the complementary material, there  is no risk of bias in the included studies |
|  | 22 | Present assessments of certainty. | Not applicable |
| **DISCUSSION** | 23 | Provide interpretation of results in context. | Sections 2.3 |
|  | 24 | Discuss limitations of evidence. | Section 2.3: “The included studies varied widely in design, populations, and measurement of variables, which limits the  comparability and generalizability of findings.” |
|  | 25 | Discuss limitations of review process. | Not applicable |
|  | 26 | Provide implications for practice, policy, and  research. | Section 2.3 |
| **OTHER**  **INFORMATION** | 27 | State whether review protocol exists and  where it can be accessed. | Not registered |
|  | 28 | Describe sources of financial or non-  financial support. | Final section: Acknowledgements |
|  | 29 | Declare competing interests. | Final section: Acknowledgements |
|  | 30 | Report availability of data, code, and other  materials. | Final section: Acknowledgements: “All data are included in  this article and its supplementary files.” |

**
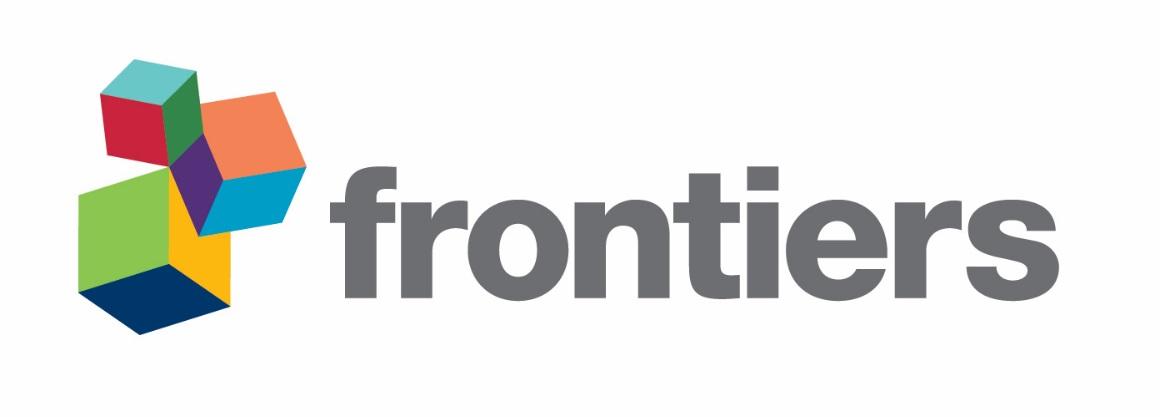
**
